# A computational exploration of bacterial metabolic diversity identifying metabolic interactions and growth-efficient strain communities

**DOI:** 10.1186/1752-0509-5-167

**Published:** 2011-10-18

**Authors:** Eleftheria Tzamali, Panayiota Poirazi, Ioannis G Tollis, Martin Reczko

**Affiliations:** 1 Computer Science Department, University of Crete, P.O. Box 2208, Heraklion, 71409, Greece; 2 Institute of Computer Science, Foundation for Research and Technology-Hellas (FORTH), N. Plastira 100, Vassilika Vouton, Heraklion, 70013, Greece; 3 Institute of Molecular Biology and Biotechnology, Foundation for Research and Technology-Hellas (FORTH), N. Plastira 100, Vassilika Vouton, Heraklion, 70013, Greece; 4 Institute of Molecular Oncology, Biomedical Sciences Research Center "Alexander Fleming", P.O. Box 74145, Varkiza, 16602, Greece; 5 Synaptic Ltd., N. Plastira 100, Vassilika Vouton, 70013, Heraklion, Greece

## Abstract

**Background:**

Metabolic interactions involve the exchange of metabolic products among microbial species. Most microbes live in communities and usually rely on metabolic interactions to increase their supply for nutrients and better exploit a given environment. Constraint-based models have successfully analyzed cellular metabolism and described genotype-phenotype relations. However, there are only a few studies of genome-scale multi-species interactions. Based on genome-scale approaches, we present a graph-theoretic approach together with a metabolic model in order to explore the metabolic variability among bacterial strains and identify and describe metabolically interacting strain communities in a batch culture consisting of two or more strains. We demonstrate the applicability of our approach to the bacterium *E. coli *across different single-carbon-source conditions.

**Results:**

A different diversity graph is constructed for each growth condition. The graph-theoretic properties of the constructed graphs reflect the inherent high metabolic redundancy of the cell to single-gene knockouts, reveal mutant-hubs of unique metabolic capabilities regarding by-production, demonstrate consistent metabolic behaviors across conditions and show an evolutionary difficulty towards the establishment of polymorphism, while suggesting that communities consisting of strains specifically adapted to a given condition are more likely to evolve. We reveal several strain communities of improved growth relative to corresponding monocultures, even though strain communities are not modeled to operate towards a collective goal, such as the community growth and we identify the range of metabolites that are exchanged in these batch co-cultures.

**Conclusions:**

This study provides a genome-scale description of the metabolic variability regarding by-production among *E. coli *strains under different conditions and shows how metabolic differences can be used to identify metabolically interacting strain communities. This work also extends the existing stoichiometric models in order to describe batch co-cultures and provides the extent of metabolic interactions in a strain community revealing their importance for growth.

## Background

In metabolic interactions also known as cross-feeding, microbial species exchange usable metabolic products arising from the metabolism of a primal nutritional resource. These metabolic products can serve as alternative, secondary resources to microbial species for obtaining their energy or composing the building blocks for biosynthesis. In nature, microbes live in communities and develop cross-feeding interactions, which provide an overall increase of nutrients and a more efficient exploitation of a given environment [1,2]. In the laboratory, evolution experiments in bacteria have repeatedly shown the emergence of cross-feeding interactions in simple environments [3-6]. Metabolic interactions can alter the biochemical phenotypes of the participating species allowing novel, unexpected phenotypes to emerge. Furthermore, the emergence of metabolic diversity and the development of metabolic interactions play an important role in the evolution of bacterial populations as they dynamically shape the growth medium. Apart from its biological significance, understanding bacterial diversity is also of great importance in areas such as food preservation and bio-degradation of pollutants as well as in human health as the extensive variability of pathogens within populations continues to threaten human life.

Several studies have worked on the characterization of microbial interactions in either synthetic or natural communities [2] and have explored the mechanisms that stabilize the emerged polymorphism in microbial populations even in simple environments. These mechanisms include the role of product inhibition in substrate competition [7,8], the spatial arrangement of interactions and dispersal in the maintenance of diversity [9-12], the existence of trade-offs between the uptake efficiencies of the primary and secondary resources [13], evolutionary criteria that allow the partial degradation of the substrate [14] and metabolic and physiological trade-offs in the absence of cross-feeding, spatial and temporal heterogeneity [15]. Game theoretical models such as the Prisoner's dilemma and the snowdrift game for pair-wise interactions and the public good games for groups of interacting individuals have been widely applied to explain stable polymorphisms, cooperative behaviors and the specific conditions that allow their establishment in natural systems [16-20]. A minimal synthetic obligatory cooperative system has also been engineered by Shou et al. [21] to study cooperation as well as the conditions that allow viability between two auxotrophic strains of yeast, each producing a substrate essential for the other. In each case where metabolic interactions are involved, the interacting species are modeled with specific properties, which allow them to share specific metabolites. Depending on the modeling approach, the fitness costs and benefits of the interactions or the nutrient fluxes are inferred from the characteristics of the individuals in order to model the interactive dynamics.

Based on existing genome-scale metabolic models, this study aims to investigate the detailed metabolic interactions, which can develop between bacterial strains as they grow in simple, single-source, batch cultures. Genome-scale metabolic models account for the inter-connectivity of metabolic pathways that utilize the environmental resources and produce energy and biomass precursors required for cellular growth. These models describe genotype-phenotype relations revealing the full extent of metabolic capabilities of genotypes under various environmental conditions and have been broadly used [22-25] with the aim of identifying essential genes or metabolites, inferring the lifestyle of an organism, investigating the evolution of the metabolic networks as well as comparing and validating the metabolic networks and designing organisms with a desirable metabolic phenotype. Although a lot of research effort has been devoted to the development of whole cell, *in-silico *genome-scale metabolic models [22,26-28], there are only a few studies of genome-scale multi-species interactions. The work of Stoylar et al. [29] is the first reported reconstruction of a dual-species stoichiometric model, which was developed in order to describe the metabolic interactions between the microbes *Desulfovibrio vulgaris *and *Methanococcus maripaludis *in methanogenic laboratory co-cultures. These two microbes develop a specific hierarchical association with each other, known as syntrophy. Although the model focuses on the central metabolism rather than the full genome, it predicts several features of co-culture growth, including the ratio of the two microbe populations in culture and the dominant electron carrier during growth. An alternative stoichiometric network analysis approach was developed by Taffs et al. [30] to describe a natural thermophilic microbial community from Yellowstone national park. Their method was also compared with previous studies and the trade-offs between the available biological knowledge of the species in a community, the tractability of the models to incorporate this knowledge and the accuracy of their predictions, were addressed. Using a set of conditionally lethal auxotrophic *E. coli *mutants, Wintermute and Silver [31] studied the pair combinations, which produced improved growth relative to their corresponding monocultures through synergistic metabolic interactions. Their model was based on the hypothesis that mutants tend to approximate the optimal wild-type flux distribution and an optimal joint metabolic flux distribution was identified to describe co-culture growth.

In this study, single, metabolic gene knockouts define the pool of bacterial strains among which potential cross-feeding interactions are examined. Based on the hypothesis that metabolic products are exchanged in a bacterial population, we previously developed a graph-theoretic approach (diversity graph) in order to map pair-wise genetic to metabolic alterations with respect to by-production and identify communities of metabolically different mutants in a given environment [32]. Different environmental conditions result in different diversity graphs and define different strain communities. In extension to our previous study several graph-theoretic measures are applied in order to reveal biologically meaningful properties, characterize the diversity graphs and allow the direct comparison of the overall metabolic variability under different growth conditions. The method can constrain the community size by determining the upper bound of the metabolic diversity in a given environment. This work also focuses on revealing the relation between metabolic difference and its evolutionary trait. Building upon the existing dynamic FBA model, which describes batch and fed-batch monocultures [33], we have also developed a metabolic model capable of describing the co-growth of different cell-competitors in a batch culture [32]. The proposed multi-competitor metabolic model assumes that cells and nutrients are distributed homogeneously throughout the growth medium and that each cell optimizes its growth depending on the availability of the substrates over time. Strain communities with more than two strains are studied in this work. The growth of strain communities are simulated with the aim to identify communities of improved growth relative to corresponding monocultures in the same batch scenario and predict the range of metabolites that are exchanged. The dependence of a strain community growth on its constituent parts is also addressed. Although strain communities are not modeled to operate towards a collective goal [29-31], such as the growth of the group, growth beneficial communities are observed as the result of metabolic interactions. We demonstrate the applicability of our approaches to the bacterium *E. coli *across different single-carbon-source conditions.

## Methods

### Modelling bacterial batch monocultures using stoichiometric models

Utilizing a genome-scale metabolic network reconstruction of an organism, constraint-based metabolic approaches model the relation between the genomic information and metabolic activity at flux level and reveal properties that cannot be predicted by descriptions of individual components [22,25]. The core assumption of constraint-based models is that the system, constrained by its stoichiometry, *S*, reaches a *steady state *(intracellular flux balancing) that satisfies the physiochemical constraints under a given environmental condition (Equation 2). Flux Balance Analysis (FBA) further assumes that a cell follows an optimization strategy in order to accomplish cellular tasks. The most commonly applied objective is the maximization of growth rate reflected in biomass production (Equation 1), which has proved to successfully describe unicellular organisms [34]. Thermodynamic constraints that determine the reversibility of the metabolic reactions and enzymatic capacity constraints have been also included to place limits on the range of possible fluxes (Equation 3).

(1) maximizeμ

(2) subjecttoSv=0

(3) $$v_{\min}\leq v\leq v_{\max}$$

Varma and Palsson [33] extend the original FBA method in order to describe batch cultures during the exponential and early stationary stage and predict the transient changes in external substrate concentrations. The batch culture consists of identical cells, which follow the same metabolic and regulatory program. Both the primal nutritional resources and the secreted metabolites are considered as substrates that can be used by cells. The model also assumes that cells and nutrients are distributed homogeneously throughout the growth medium.

The initial concentrations of the substrates (*exC*_*0*_) are given as well as the initial biomass concentration (*b*_*0*_) of the bacterial population. The whole time regime is divided into time intervals *δ t *where intracellular steady-state is assumed. At each time interval the flux distribution, *v*, which optimizes the growth rate *μ *is calculated by solving the optimization problem described in Equations 1, 2, 3 and 6. The biomass and substrate concentrations are updated at each time interval as shown in Equations 4 and 5, respectively. The flux $v_{ex}^{j}$ of an exchange reaction *j *is positive if the substrate corresponding to reaction *j *is produced and negative when it is consumed. Equation 6 corresponds to the uptake bounds of the exchange fluxes, which are determined by the availability of substrates at each time point.

(4) $$b\left[t\right]=b\left[t-\delta t\right]e^{\mu \cdot \delta t}$$

(5) $$exC\left[t+\delta t\right]=exC\left[t\right]-v_{ex}\frac{b\left[t\right]}{\mu }\left(1-e^{\mu \cdot \delta t}\right)$$

(6) $$v_{\min}^{ex}=-\frac{exC}{b\cdot \delta t}$$

In this work, the batch monoculture simulations of the single-gene knockout *E. coli *strains are performed using the *in silico *metabolic network of *E. coli *(*i*JR904) by Reed et al. [35], which includes 904 genes and 931 biochemical reactions. Of the 68 single-carbon source conditions described in the work of Covert et al. [36], 58 carbon sources are examined in this work since 10 of the 68 conditions did not allow the growth of any mutant or wild-type. The initial biomass concentration is set to 0.003 gDW/lt. The initial concentration of the carbon source is set to 10 mmol/lt. Oxygen, ammonia and other important inorganic metabolites are assumed to be in excess in the growth medium. The initial bounds of the uptake rates are also set in accordance to the work of Covert et al. [36]. The time resolution (*δt*) is 0.1 h. Maximization of the growth rate is used as the objective function of the optimization problem. A second optimization problem is also applied to minimize the enzymatic cost expressed by the absolute flux values under the constraint that the cell continuously operates at the maximum growth rate [37]. The glpk solver [38] is used for solving the linear programming problems. Simulations are performed using the COBRA toolbox [39].

### Modelling bacterial batch co-cultures using stoichiometric models

Building upon the existing dynamic FBA model, which describes batch (and fed-batch) monocultures [33], we have developed a metabolic model capable of describing the co-growth of different cell-competitors in a shared batch culture [32]. As in monocultures, the proposed model assumes that cells and nutrients are distributed homogeneously throughout the growth medium and that the primal nutritional resources as well as the secreted metabolites can be used by the cells, which allow the development of cross-feeding interactions within the heterogeneous population. Contrary to previous stoichiometric models [29-31], the different cells are not modeled to operate towards a collective goal, such as the maximization of the community growth. Instead, each different cell is assumed to maximize its individual growth.

The initial biomass concentration ($b_{0}^{i}$) of each stain-competitor as well as the initial concentrations of the substrates (*exC*_0_) in the growth medium are given. At each time interval the flux distribution (*v*_*i*_), which optimizes the growth rate *μ*_*i *_is calculated for each strain *i *independently solving the optimization problem described by the Equations 1, 2, 3 and 9. Equations 7 and 8 show how the biomass concentration of each strain (*b*_*i*_) and the substrate concentrations (*exC *) of the medium are updated at each time interval. The current availability of the substrates and the bacterial population determine the uptake bounds of the exchange fluxes as shown in Equation 9. The simulation terminates when none of the strain-competitors can grow further in the shaped medium, which usually corresponds to the phase of nutrient depletion. If the population consists of identical cells, the multi-competitor metabolic model is reduced to the existing dynamic FBA model [33]. It should be noted that competitors in our model can also represent different species, assuming the corresponding genome-scale metabolic networks are available.

(7) $$b_{i}\left[t\right]=b_{i}\left[t-\delta t\right]e^{\mu_{i}\cdot \delta t}$$

(8) $$exC\left[t+\delta t\right]=exC\left[t\right]-\underset{i}{\sum }v_{ex}^{i}\frac{b_{i}\left[t\right]}{\mu_{i}}\left(1-e^{\mu_{i}\cdot \delta t}\right)$$

(9) $$v_{\min}^{ex}=-\frac{exC}{\delta t\cdot \underset{i}{\sum }b_{i}},\;\forall i$$

In this work, communities consisting of two or more *E. coli *strains are simulated under different carbon conditions in batch co-cultures, utilizing the aforementioned metabolic model. To allow comparison between monocultures and co-cultures, the growth settings of these simulations are the same with those described for monocultures and the initial biomass concentration of 0.003 gDW/lt is equally distributed to the different strain populations, unless stated otherwise. The heterogeneous population is mainly studied with respect to its growth benefit and the involved cross-feeding interactions. The metabolic interactions between strains can be identified through the flux time profiles of the exchange reactions when co-growing on a given environment, whereas the concentration of the nutrients indicates the exploitation (if any) of the common growth medium. The single-carbon-source conditions that are examined in this study for the identification of efficient strain communities include *glycolate*, *acetate*, *glycine*, *glucose, pyruvate *and *melibiose*.

### Diversity graph construction

Metabolic interactions that involve the exchange of intermediate metabolic products are assumed to occur only if the metabolic capabilities of the members of the bacterial population differ with respect to by-production. Based on this assumption, a graph representation is constructed in order to reflect the metabolic variability with respect to by-production within a pool of genetically different cells [32].

Specifically, single-gene knockouts are applied on the *E. coli *metabolic network [35] to generate the pool of mutants. The growth of each *E. coli *strain in a single-carbon-resource batch culture is simulated using the dynamic FBA method described previously for monocultures. The nodes of the metabolic diversity graph correspond to viable strains in a given growth condition. For each viable strain *i*, we construct a feature vector, which consists of the maximum concentration values (${}_{\max}C_{i}^{s}$) of each secreted metabolite (*s *) that is produced during its growth on a specific single-carbon environment. This vector is used as a metabolic blueprint for the potential interactions of the strain. As shown in Equation 10, for each pair of strains the relative difference of their feature vectors is calculated and the maximal difference over all byproducts is used as a weight for the interaction between them. According to the above definition, the edge weights in diversity graphs take values between 0 and 1, where weights equal to 0 imply that the two strains are identical regarding by-production and weights equal to 1 imply that (at least) one of the two strains provides a novel byproduct to the other strain. As the metabolic products of a strain depend on the growth environment, a different diversity graph is constructed for each single-carbon environment.

(10) wij=maxs(|Cmaxis−Cmaxjs|max(Cmaxis,Cmaxjs))where Cmaxis=max(Cis(t))

The by-production efficiency is defined as the ratio of by-production intake rate to primal carbon resource uptake rate. As shown in Additional file 1: Supplement DG, the edge weights in diversity graphs also represent the maximal relative difference of by-production efficiency between strain pairs. This indicates that the diversity graph is independent of the initial concentration of the primal source, which allows a unique graph representation of the metabolic differences of the mutants for a given growth condition. Furthermore, it implies that the diversity graph can also be estimated by the original, single time-step FBA model, which accelerates the reconstruction of the diversity graphs.

### Diversity graph analysis

Several graph-theoretic measures are applied in the diversity graphs to allow the direct comparison of the overall metabolic variability of *E. coli *strains under different carbon-source conditions and reveal biologically relevant properties such as the association between metabolic difference and its evolutionary trait. In this study, the graph-theoretic analysis is performed on the (original) weighted representations of the diversity graphs.

The definitions of the graph-theoretic measures used in this study, which include strength centrality, assortativity and clustering coefficient are given in Additional file 2: Supplement SA.

### Identification of strain communities

**Figure 1:**
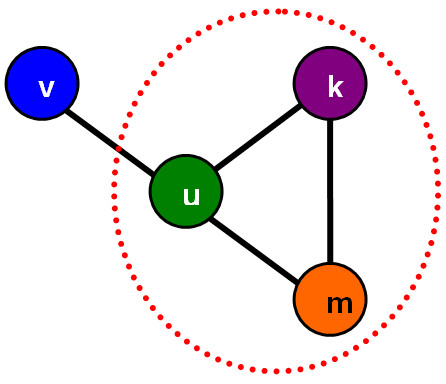
**Community in a simplified diversity graph**. A simple diversity graph consisting of the strain-nodes *v*, *u*, *k *and *m*. Edges are assigned between metabolically different strains. As strains *v *and *k *are metabolically similar, either *v *or *k *can form a community with *u*, both are metabolically redundant. On the other hand, the strains *u*, *k *and *m *are different from each other forming a community of size 3. The identification of strain communities is reduced to identification of cliques in a diversity graph.

The composition of a metabolically interacting strain community can be assumed to consist of individuals with the potential to differently shape the given environment and provide each other with products of their metabolism. To ensure compositions of strains with different metabolic capabilities, each strain must be different from all the other strains. Therefore, compositions of strains with different metabolic capabilities correspond to cliques (complete sub-graphs in which all nodes are connected with each other) in a diversity graph (Figure 1). Furthermore, the maximum clique size that can be found in a given diversity graph actually determines the upper bound of the metabolic diversity that can emerge in a population in a given growth condition under the specific genetic perturbations. The cliques are identified using the binary representation of the diversity graph, which is produced using a threshold of 0.6. This threshold value indicates that connected mutants are relatively highly different in terms of their metabolic characteristics. In this work, cliques are identified using efficient, exact methods that have been proposed elsewhere [40].

Because of the genetic robustness and redundancy that is inherent in metabolic networks under single gene knockouts [41-46], there are only few strains with novel metabolic capabilities. This metabolic redundancy is reflected in the diversity graphs and allows significant compression in the size of the graph. All strains with the exact same metabolic capabilities regarding by-production under a given initial environmental condition can be grouped together. More precisely, the structural compression maps all metabolically redundant nodes of the same connectivity (structurally identical) onto a super node. This compression can be used to accelerate the clique identification problem and it also allows the graph to be visualized by highlighting the nodes and interactions that actually produce the metabolic diversity of the system. Although cliques are found using the compressed graph, they are then decompressed to be used in simulations.

### Growth efficiency

In this work, growth efficiency concerns the ability of a cell population to maximize its growth performance in a batch culture given an initial limited amount of resources. The growth performance of a cell population is measured with respect to the maximum (endpoint) biomass concentration. If the cellular population consists of different cells, then the growth performance of the heterogeneous population corresponds to the total biomass concentration of the group, which is determined by the summation of the biomass concentration of each of the members of the group.

In cases of homogeneous, monoclonal cell populations, individual growth performances are compared with each other under the same initial conditions to determine the population with superior performance. To quantitatively describe superior performance in heterogeneous populations, we define the *absolute *and the *relative *benefit. If the group performance of an heterogeneous cell population, *g*, is superior to the performance of any (wild type or mutant) monoculture, *m*_*i*_, then the heterogeneous community under study is beneficial. We term this benefit 'absolute'. However, the condition of 'any' can be relaxed, so that the growth performance of a heterogeneous community is compared to the homogeneous performances of its corresponding community members. In this case we call the benefit 'relative'. The relative benefit indicates whether there is *group benefit*, that is, whether the group is more efficient as a whole than the efficiency of any of its members when functioning as individuals.

To measure benefit we use Equation 11. Absolute and relative benefits differ with respect to whether the *i *elements correspond to all strains or only the members of the specific group. If the co-culture does not show improved growth relative to corresponding monocultures then the benefit is negative.

(11) $$\text{benefit}=\frac{\left(g-\max_{i}\left(m_{i}\right)\right)}{\max_{i}\left(m_{i}\right)}$$

## Results

For simplicity reasons, *E. coli *strains are henceforth named after the name of the gene that has been deleted.

### Centrality measures reflect the metabolic redundancy of *E. coli *and reveal unique phenotypes for each growth condition

Diversity graphs are constructed in order to represent pair-wise differences in by-production between *E. coli *strains (see Methods). Each single-carbon-source growth condition corresponds to a different diversity graph. The node centrality [47,48] is a local measure, which expresses the importance of a node in a graph with respect to its connections. In the diversity graphs, a highly central node indicates a mutant of considerably different (unique) metabolic capabilities regarding by-production than the rest of the mutants. On the other hand, a non-central node corresponds to a mutant which exhibits similar (redundant) metabolic capabilities with most of the mutants of the graph.

The strength distribution of each diversity graph (see Additional file 2: Supplement SA) shows that the majority of nodes (above 80%) are non-central and only a few (below 10%) have strength centrality greater than 0.9, whereas nodes with intermediate centrality values are less than 10% of the graph. The network centrality measure (see Additional file 2: Supplement SA) also indicates that most diversity graphs are highly centralized. These observations imply high metabolic redundancy and the presence of only a few mutants with unique metabolic capabilities.

Furthermore, as shown in Additional file 2: Supplement SA the high values of the network clustering coefficient, which is defined in [49] to reflect the cliquishness of a neighborhood in a graph as well as the dependence between clustering coefficient and centrality, show that central nodes are highly connected with each other forming a highly clustered area in the graph and that the redundant mutant group is part of this highly connected area. As a result, central mutants and their connectivity reflect the metabolic diversity regarding by-production of *E. coli *strains and play an important role in the formation of communities, which consist of metabolically different strains.

The diversity graph of *adenosine *comprises an exception, as it exhibits a broader strength centrality distribution than the rest carbon-source graphs and the lowest network strength-centrality of value close to 0.6, thus implying a system of higher metabolic variability. The diversity graphs of *acetate *and *glycolate*, on the other hand, are star networks consisting of exactly one central node, which indicates growth conditions with very high metabolic redundancy. This finding is expected as these two carbons are also by-products of other carbon-source growth conditions. Specifically, *acetate *and *glycolate *are the most frequently observed by-products across the carbon-conditions tested (see Additional file 2: Supplement SA) and when *glycolate *is the main source, the central mutant of the graph is the only mutant that produces *acetate *and vice versa.

Genome-scale deletion phenotype data for the bacterium *E. coli *have shown that the metabolic network is inherently robust to genetic perturbation and environmental changes with respect to cell viability and maintaining vigorous growth [50]. In addition to these observations, the centralized topology of diversity graphs further implies that the cellular response to single-gene knockouts rarely affects by-production.

**Figure 2:**
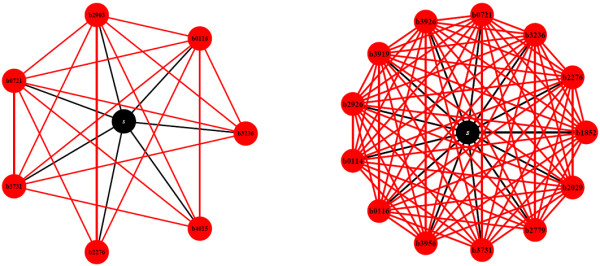
**Examples of Diversity Graphs**. The binary representation of two diversity graphs is visualized after structural compression, which maps all metabolically redundant nodes of same connectivity onto a super-node. The super node is depicted with black color and labeled with the letter *s*. The highly central nodes are depicted with red color and the names of the corresponding deleted genes are shown. All highly central nodes are connected with the super node. (Left) The diversity graph of *pyruvate*. 7 highly connected nodes are identified. 373 nodes comprise the super-node. (Right) The diversity graph of *D glucose*. The graph consists of 13 highly connected nodes and the super-node includes 373 nodes.

The binary graph representations of two examples (*pyruvate *and *glucose*) after structural compression that maps all metabolically redundant nodes of same connectivity onto a super node (see Methods) are shown in Figure 2. This mapping highlights the metabolically unique (highly central) strains and their connections. The metabolic reactions as well as the metabolic subsystem in which the deleted genes participate can be found in Table 1 for most of these highly central strains.

**Table 1:** Metabolic and Conservation (ERI) Information of the deleted genes in consistently appearing *E. coli *strain-hubs.

Gene	Metabolic reactions	Metabolic sub-systems	ERI
b2276	'NADH dehydrogenase ubiquinone 8 35 protons' 'NADH dehydrogenase menaquinone 8 2 protons' 'NADH dehydrogenase demethylmenaquinone 8 28 protons'	'Oxidative Phosphorylation'	0.59
b3731	'ATP synthase four protons for one ATP '	'Oxidative Phosphorylation'	0.78
b2779	'enolase'	'Glycolysis-Gluconeogenesis'	0.97
b3236	'malate dehydrogenase'	'Citric Acid Cycle'	0.81
b0116	'2 Oxogluterate dehydrogenase' 'Glycine Cleavage System' 'pyruvate dehydrogenase'	'Citric Acid Cycle' 'Folate Metabolism' 'Glycolysis-Gluconeogenesis'	0.84
b2926	'phosphoglycerate kinase'	'Glycolysis-Gluconeogenesis'	0.97
b0721	'succinate dehydrogenase'	'Citric Acid Cycle' 'Oxidative Phosphorylation'	0.28
b0114	'pyruvate dehydrogenase'	'Glycolysis-Gluconeogenesis'	0.38
b3956	'phosphoenolpyruvate carboxylase'	'Anaplerotic reactions'	0.34
b2551	'D alanine transaminase' 'alanine transaminase' 'glycine hydroxymethyltransferase' 'Threonine Aldolase'	'Cofactor and Prosthetic Group Biosynthesis' 'Cofactor and Prosthetic Group Biosynthesis' 'Glycine and Serine Metabolism' 'Threonine and Lysine Metabolism'	0.97
b3919	'triose phosphate isomerase'	'Glycolysis-Gluconeogenesis'	0.94
b0529	'methenyltetrahydrofolate cyclohydrolase' 'methylenetetrahydrofolate dehydrogenase NADP'	'Folate Metabolism' 'Folate Metabolism'	1.00

### The upper bound of metabolic diversity

**Figure 3:**
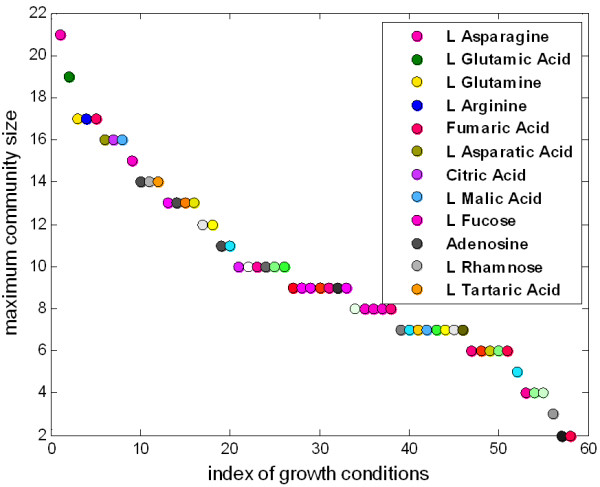
**Maximum strain-community size**. The maximum community size varies between 2 and 21 depending on the carbon-growth condition where the minimum value is observed in the diversity graphs of *acetate *and *glycolate *and the maximum value corresponds to *L- asparagine*. The carbon conditions in which the 12 largest community sizes correspond are shown.

Polymorphic communities consisting of strains with different metabolic capabilities correspond to cliques in the diversity graph (see Methods). The maximum clique size of a diversity graph reflects the actual number of the different metabolic patterns regarding by-production and determines the upper bound of the metabolic diversity that emerge in a given growth condition under single-gene knockouts. As shown in Figure 3, the maximum clique size varies between 2 and 21. The minimum value is observed in the diversity graphs of *acetate *and *glycolate*, due to their star topology and the maximum value corresponds to the carbon condition *L- asparagine*.

Since the metabolic variability is defined with respect to by-production, the total number of the metabolites that are by-produced bounds the maximum metabolic variability that can emerge in a given growth condition. Theoretically, if *N *is the number of by-products and if we consider a metabolite as only produced or non-produced by a strain, ignoring its concentration, then the total number of different subsets is 2^*N*^. However, as shown in Additional file 2: Supplement SA the number of different subsets, which define the maximum clique size in a given growth condition is considerably less than 2^*N*^. This is due to the fact that the metabolic pathways are coupled so that activating a pathway that leads to the production of a metabolite affects the production of other metabolites as well.

Cliques are found using the binary representation of the diversity graphs, which is produced using a threshold of 0.6 in the corresponding weighted graph (see Methods). However, as shown by the edge weight distributions (Additional file 2: Supplement SA), the resulting graphs are very robust with respect to the choice of the threshold value.

### The evolutionary trait of metabolic diversity as reflected in assortativity coefficient

Two strains are heavily connected in the diversity graph if they are highly different with respect to their by-products. The assortativity coefficient [51-53] is used in this work to explore the relation between metabolic difference and its evolutionary trait. Specifically, an index is assigned to each strain in the diversity graph to reflect how conserved the corresponding deleted *E. coli *gene is across different organisms. In this study, we use the Evolutionary Retention Index (ERI) to express gene conservation as introduced in the study of Gerdes et al. [50]. A graph is then characterized as assortative (or disassortative) by ERI if strains of similar (or dissimilar) conservation value in their corresponding genes are preferably connected with each other.

As shown in Additional file 2: Supplement SA, the diversity graphs of most carbon conditions tend to be disassortative by ERI. This implies that deletions of non-conserved genes tend to generate strains that are metabolically similar with each other regarding their by-products and only if one of the two strains is related with deletion of a highly conserved gene, are more likely to be metabolically different. This further indicates an evolutionary difficulty towards the establishment of polymorphism. Furthermore, considering that most edges in the diversity graph are between metabolically redundant and metabolically unique strains, this finding also implies that these two classes of strains tend to correspond to deletions of genes with different conservation values.

### Consistent metabolic behaviors across growth conditions

**Figure 4:**
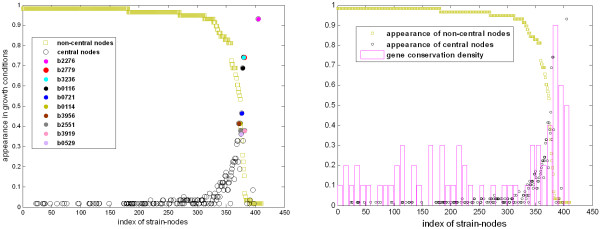
**Consistent metabolic behaviours and their evolutionary trait**. (Left) The frequency of appearance of the central and the non-central mutants over the 58 different carbon-source conditions (empty black circles and empty green squares respectively). Redundant nodes in some growth conditions can be central mutants in other conditions. The most frequently-appearing highly central mutants are highlighted (filled colored circles). (Right) The gene conservation density is shown for each strain. Environmental-invariant strain-hubs correspond to gene deletions in more conserved genes relative to environmental-specific hubs. Similarly, environment-invariant redundant strains correspond to deletions in less conserved genes than the environment-specific redundant strains. This finding implies that a polymorphic strain community is more likely to evolve if it consists mainly of environment-specific strains as they are evolutionary more stable.

It was previously shown that the diversity graphs are highly centralized consisting of many nodes of low centrality and a few nodes of high centrality. The frequency of appearance of the central and the non-central nodes across the carbon conditions is shown in the left panel of Figure 4 (empty black circles and the empty green squares respectively). Central nodes represent strains of unique metabolic capabilities regarding by-production and comprise the core of strain communities. A subset of these structurally important mutants consistently appears in most of the examined growth conditions, as shown in the left panel of Figure 4 (filled colored circles). The reactions and the metabolic subsystem, in which the deleted genes of the most frequent central strains participate, are presented in Table 1. On the other hand, non-central nodes correspond to strains of common, redundant metabolic capabilities regarding by-production. As shown in Figure 4, environmental-specific redundant mutants often have a central role in other growth conditions and vice versa. Intuitively, one would expect that the frequently-appearing unique phenotypes might reflect deletions of essential and evolutionary conserved genes across different organisms, whereas the opposite must be true for frequently-appearing redundant phenotypes. Next, we investigate this hypothesis.

We define the gene conservation density as follows. We use a resolution step of 10 mutants and measure the percentage of mutants, in which the corresponding deleted genes have a conservation value (ERI) above 0.7. As shown in the right panel of Figure 4, the environment-invariant hubs correspond to deletions of relatively more evolutionary conserved genes than the environmental-specific hubs. In particular, approximately 60% of the consistently appearing central mutants are related to the knockout of a highly conserved gene in contrast to the environment-specific mutants of which approximately 30% correspond to highly conserved genes. The correlation coefficient between the gene conservation density and the frequency of appearance of the corresponding mutants is 0.6826 (p-value < 0.0025). On the other hand, consistently redundant mutants are mostly derived from deletions of non-highly conserved genes. Specifically, 50-90% of the genes involved in the environment-specific mutants are evolutionary conserved, whereas only 20-30% of the genes are conserved in the environment-invariant redundant mutants. The correlation coefficient between the gene conservation density and the frequency of appearance of the corresponding metabolically redundant mutants is -0.8224 (p-value < 10^-10^).

To conclude, most environment-invariant central mutants correspond to deletions of highly conserved genes. This observation seems controversial to the evolution of polymorphism, which necessitates the presence of unique phenotypes, because if a gene is evolutionary conserved, the mutant derived from its deletion is expected to be evolutionary unstable. However, it suggests that among all potential communities only those consisting mainly of mutants that are environment-specific are likely to evolve.

### Beneficial interactions and growth-efficient strain communities

Previous results based on an exhaustive, computational evaluation of all pairs consisting of the wild-type and a single-gene knockout mutant have revealed several pairs of improved growth relative to the corresponding monocultures [54]. An update of these results is shown in Additional file 3: Supplement FA. In this work, the diversity graph construction is used to identify compositions of strains with different metabolic capabilities regarding by-production. Using the developed multi-competitor metabolic model (see Methods), the growth of these strain communities is simulated under several single-carbon conditions, which include *glycolate*, *acetate*, *glycine*, *glucose, pyruvate *and *melibiose*. As defined in the Methods section, the term relative benefit is used when the growth performance of the group is compared with the growth performance of the monocultures of each individual member mutant, whereas the term absolute benefit is used when the co-culture is compared with each monoculture in the diversity graph.

**Figure 5:**
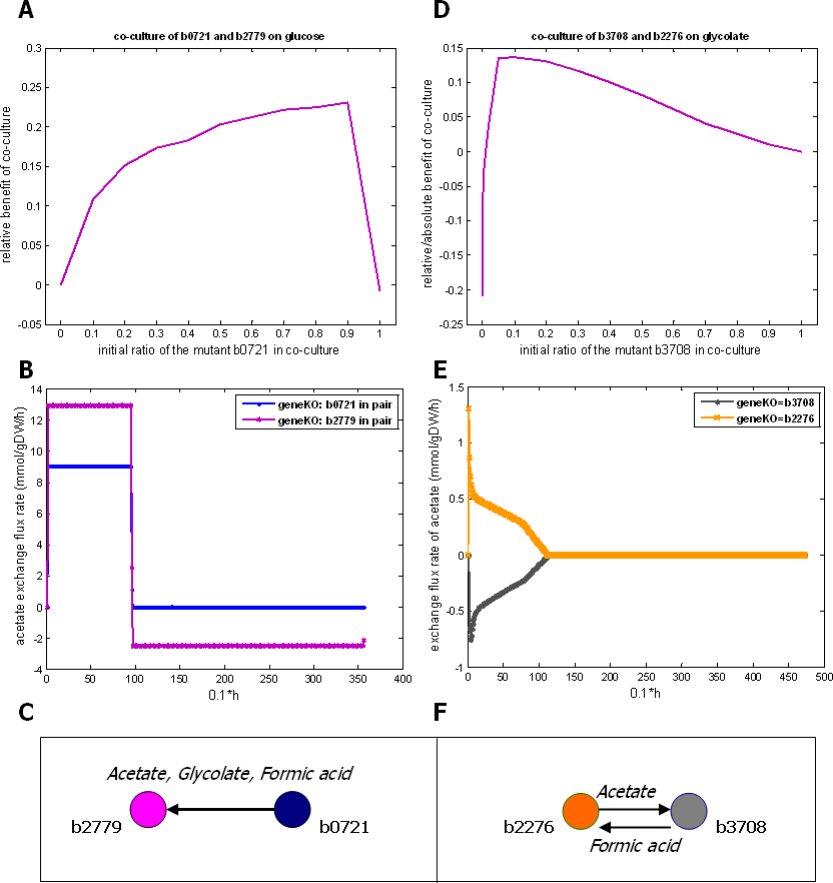
**Cross-feeding interactions improve growth**. (A) Predicted relative benefit of the co-culture consisting of the mutant b2779 and b0721on *glucose *for various initial population ratios. Maximum relative benefit (23%) is achieved with an initial population composition of 1:9 for b2779:b0721. The mutant b0114 is the most efficient monoculture on *glucose *and no strain community has been found to perform better than this monoculture. Thus, we refer to relative benefit here. (B) The *acetate *exchange flux profiles of each mutant in the co-culture of b2779 and b0721 as they co-grow on *glucose*. When *glucose *is exhausted, the produced *acetate *is exclusively consumed by the mutant b2779. (C) A schematic diagram of the cross-feeding interactions between the mutant b0721 and b2779 on *glucose *is shown. The mutant b0721 plays the role of a provider. (D) Predicted relative (and absolute) benefit of the co-culture consisting of the mutant b2276 and b3708 on *glycolate *for various initial population ratios. Maximum group performance (equals to 14%) is observed when the two mutants exhibit equal final frequency in the population, which is achieved with an initial population composition of 1:9 for b3708:b2276. The mutant b3708 is metabolically similar to the wild-type. (E) The *acetate *exchange flux profiles of each mutant in the co-culture of b2276 and b3708 as they grow on *glycolate*. As long as the metabolism of *glycolate *takes place, *acetate *is produced by b2276 and consumed by b3708. (F) A schematic diagram of the cross-feeding interactions between the mutant b2276 and b3708 on *glycolate *is shown.

The growth simulations reveal the existence of several beneficial strain communities, which show improved growth relative to their corresponding monocultures. Beneficial metabolic interactions can be either bi-directional, where the exchange of essential nutrients takes place in both directions, or they can be unidirectional where only one benefits from the coexistence and the other plays the role of a mere provider, an altruist. An example of pure altruism is observed when the strains b0721 and b2779 co-grow on limited *glucose*. Both these mutants consistently appear as central nodes in the diversity graphs (Table 1). When b0721 grows on *glucose*, it produces the metabolites *acetate, glycolate *and *formic acid*, which apart from *glycolate *it is incapable of consuming. The exchange flux rate time profiles show that the mutant b2779 in co-culture exploits the available metabolites (Figure 5B and Additional file 3: Supplement FA). In particular, *acetate *and *formic acid *are consumed after *glucose *is exhausted, while *glycolate *is consumed in parallel with the consumption of *glucose*. The involved cross-feeding interactions are illustrated in Figure 5C. The growth performance of the pair is examined under different initial population ratios as well. As shown in Figure 5A, maximum group performance of the co-culture is achieved with an initial population composition of 1:9 for b2779:b0721, where the relative benefit equals to 23%.

Another beneficial community is the co-culture of b2276 and b3708 (or the wild-type) on limited *glycolate*. This pair in particular performs better than any monoculture in the diversity graph exhibiting absolute benefit equal to 8% (Figure 5D). The mutant b2276 is the most frequently appearing mutant-hub across all carbon-source conditions (Table 1). When this mutant grows as monoculture on limited *glycolate*, it exhibits poor growth performance (~20% reduction) compared to the growth performance of the wild-type population. As illustrated in Figure 5F, the metabolic interactions are bi-directional and correspond to the exchange of *acetate *and *formic acid*. These products are consumed simultaneously with the metabolism of *glycolate *(Figure 5E and Additional file 3: Supplement FA). An increase in the maximum growth rate of both mutants is observed during the by-product exploitation period (Additional file 3: Supplement FA). The growth performance of the pair is also examined under different initial population ratios. As shown in Figure 5D, maximum group performance (of absolute benefit equal to 14%) is observed with an initial population composition of 1:9 for b3708:b2276, which occurs when the two mutants exhibit equal final frequency in the population (Additional file 3: Supplement FA).

Efficient strain communities are not limited to pairs. An example is the triplet consisting of the strains b2903, b3403 and b0721 when growing on limited *pyruvate*. The nutrients that are exchanged between them include *glycine*, *glycolate*, *acetate *and *formic acid*. The corresponding flux profiles of each strain in the community are shown in Additional file 3: Supplement FA. The specific community is 1.4% more efficient than the most efficient monoculture, which is the strain b3403. The by-products *glycolate *and *glycine *are consumed respectively by the strains b0721 and b3403 during the metabolism of *pyruvate*. This parallel consumption of the by-products with the main resource increases the maximum growth rates of both mutants (Additional file 3: Supplement FA). *Acetate *and *formic acid*, on the other hand, are consumed after the depletion of *pyruvate*. Furthermore, beneficial cross-feeding interactions can occur either directly or indirectly. The indirect metabolic interactions imply that a bacterial community can be beneficial even if not all of its pair-wise relations involve the exchange of nutrients, which demonstrates the importance of studying group-wise metabolic variability. An example is observed in the triplet b0721, b4015 and b0728 when growing on limited *pyruvate *(Additional file 3: Supplement FA). The mutants b0721 and b4015 do not interact. Furthermore, each of the constituent pairs of the triplet is non beneficial (negative relative benefit). However, the coexistence of all three strains becomes beneficial with relative benefit equal to 12.3%.

Simulations show that no strain community exhibits improved growth unless metabolic interactions are involved within the community. Thus, although metabolic interactions are possible to take place without a beneficial outcome, they are indispensable within strain communities in order to perform efficiently under conditions of resource competition.

Beneficial communities of relative benefit performing better than their corresponding monocultures are found in all conditions we have examined (see Additional file 3: Supplement FA). However, strain communities of superior growth performance exhibiting improved growth relative to any monoculture in the diversity graph (absolute benefit) are less frequent. The existence of efficient strain communities of improved growth relative to any tested monoculture implies that in specific growth conditions, the involved metabolic pathways are coupled in a way that a single optimal mutant is incapable of fully utilizing the environment. In that case, among all single-gene knockout mutants simulated to grow as monocultures, none is capable of combining maximum ability to metabolize the main source with maximum ability to metabolize essential products of the metabolism in the specific conditions.

### Metabolic opportunities and growth predictability

The growth performance of a strain community depends on the growth properties and metabolic capabilities of the strains (e.g. their growth rates and ability to metabolize specific metabolites) as well as the interactions between them. Metabolic interactions can alter the biochemical phenotypes of the participating strains allowing novel, unexpected phenotypes to emerge.

A strain community is considered unexploited regarding by-production if cross-feeding interactions either do not occur or do not fully exploit the metabolites produced in co-culture. An unexploited community gives the opportunity to a new strain to utilize the available metabolites. One such example was discussed previously and concerns the community consisting of the strain b0721, b4015 and b0728 when growing on limited *pyruvate*. Among these strains, b0721 and b4015 do not interact, while the strain b0728 both exploits the metabolites produced by these strains and provides by-products that these two strains can metabolize.

**Figure 6:**
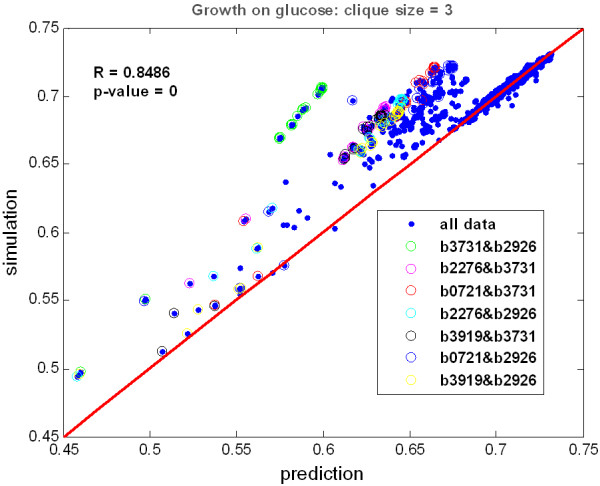
**Strain communities depend on their constituent parts**. The simulated growth performance of all the strain triplets (simulation) that are identified in the diversity graph of *glucose *with respect to the mean of the performances of the strain pairs (prediction) is shown. The correlation coefficient equals to 0.8486 (p-value = 0). The points that lie above the line where predicted growth equals simulated growth (shown in red) correspond to unexploited strain pairs and are highlighted with different colors. These strain pairs, in particular, are edges between highly central nodes.

Interestingly, the growth performance of a strain community of any size linearly depends on the mean of the growth performances of its constituent strain pairs (Figure 6 and Additional file 3: Supplement FA) as long as each of these pairs interact. In other words, under these constraints, the community can be considered to consist of non-interacting, independent sub-communities, so that by knowing the growth performances of these sub-communities we can predict the growth of the whole community. However, as more complex metabolic interactions are developed within polymorphic populations, new phenotypes that were not expected before are likely to emerge. As the composition of the communities becomes more complex, consisting of more specialized mutants and allowing more obligatory relations to be developed between them, predictability of the growth performance of the communities from their simplest constituents vanishes [55].

## Discussion and Conclusions

Most microbes live in communities and usually rely on metabolic interactions to increase their supply for nutrients and better exploit a given environment [2,56]. Several interesting examples presented in [55] show synergistic interspecies interactions developed in oral microbial communities. In addition, long-term evolutionary experiments on initially monoclonal bacterial populations have shown that the bacterial population rapidly evolves to polymorphic populations even in simple, homogeneous, single-limited resource laboratory environments. The evolved strains are considerably different from each other with respect to their metabolic capabilities and it is shown that metabolic interactions between them take place [3]. The emergence of metabolic diversity and the development of metabolic interactions play an important role in the evolution of bacterial populations as they dynamically shape the growth medium.

Using a graph representation, this work has first explored the metabolic variability regarding by-production among single-gene knockout *E. coli *strains in various single-carbon source conditions. The diversity graph maps in a pair-wise manner the genetic to metabolic variability with respect to by-production and allows the identification of strain communities (corresponding to cliques) with the potential to exchange products of their metabolism in a given growth condition. A different diversity graph has been constructed for each single-carbon environment. The properties of the diversity graphs reflect the inherent high metabolic redundancy of the cell to single-gene knockouts, reveal mutants of unique metabolic capabilities regarding by-production and show an evolutionary difficulty towards the establishment of polymorphism. Furthermore, findings of this work suggest that polymorphic communities consisting of strains specifically adapted to a given condition are more likely to evolve.

In addition to the diversity graph and its structural analysis, a developed genome-scale metabolic model has been used to describe the co-growth of different cells in a batch culture. The proposed multi-competitor metabolic model was based on the existing dynamic FBA model [33], which successfully describes monoclonal bacterial population in a batch culture. Contrary to previous multi-cellular stoichiometric models [29-31], the different cells have not been modeled to operate towards a collective goal, such as the optimum growth of the group. However, the proposed model assumes that the different cells grow in a spatially homogeneous environment sharing nutrients and maximizing their growth rates in accordance to their metabolic capabilities. Under these assumptions, the model has been utilized in order to test the hypothesis of whether strain communities can be more efficient than their corresponding monocultures and predict the range of metabolites that are exchanged. The growth simulations revealed many strain communities that were beneficial, namely performed better as a whole than their individual parts. Moreover, the existence of efficient strain communities performing better than any examined monoculture were also identified in some growth conditions. This finding implies that in some growth conditions, the involved metabolic pathways are coupled in a way that a single optimal mutant is incapable to fully utilize the environment. In addition, it was observed that metabolic interactions took place without necessarily leading to a beneficial outcome. However, they were indispensable within strain communities in order to perform beneficially under conditions of resource competition. In other words, the metabolic interactions are the necessary (but not sufficient) condition in order for a group to perform beneficially. Since the initial population frequency of the competitors as well as the amount of the main source that is initially provided for growth play an important role in the growth performance of the communities as they determine the partitioning of the resources, it is expected that the beneficial communities are not limited to our observations even within the given search space.

The construction of diversity graphs as well as the evaluation of the *E. coli *strain communities were built upon the genome-scale metabolic network reconstruction of *E. coli *[35] as well as the constraints and assumptions of the existing dynamic FBA model [33]. The predictive accuracy of the proposed methodology depends on the accuracy of the genome-scale metabolic reconstructions and their reliable prediction of the transport fluxes under genetic perturbations and growth conditions [57-60]. In this study, all simulations were performed on a single-limited carbon resource. However, during growth several metabolites can be produced, which serve as secondary resources for growth. Thus, the initially simple environment becomes a mixed-substrate growth medium where metabolic interactions can take place. In batch cultures containing a mixture of carbon sources, microbial cells utilize the substrates either sequentially, a phenomenon known as *diauxic *growth, or simultaneously depending on the medium [61,62]. The metabolic model of Varma and Palsson [33] for monocultures accurately predicts the reutilization of *acetate *in *glucose *batch cultures. In our simulations, both sequential and simultaneous substrate consumption examples are predicted, the accuracy of which remains to be experimentally validated. Efforts to model mixed-substrate growth based on the FBA framework have been proposed such as the work of Beg et al. [63], which proposes an improved FBA model that incorporates a solvent capacity constraint for the enzymes inside the cytoplasm. Alternatively, the incorporation of regulatory constraints on the FBA model has been also suggested [36,64,65] to improve the accuracy of substrate uptakes in a complex medium.

Other sources of genetic to metabolic diversity such as multiple gene deletions or differential expression of certain genes can also contribute as nodes to the graph reconstruction and as potential strain-competitors in communities, beyond the single-gene knockout strains. Metabolic interactions across different species can also be analyzed under the framework proposed here, as more genome-scale metabolic network reconstructions are available. The results presented in our work are not primarily focused on evolutionary stable communities that may arise from cross-feeding. The method aims to identify and describe metabolic interactions between strains that co-exist in a batch culture. Further analysis regarding the evolutionary stability of the strain communities and their survival from 'cheats', who only gain the benefit from others [11,13,19,66-69], is particularly interesting.

## Competing interests

The authors declare that they have no competing interests.

## Authors' contributions

ET and MR conceived the study, IGT conceived the graph theoretic analysis, PP and MR participated in the coordination of the study and contributed to the interpretation of data and to the writing of the paper. ET conducted all experiments and wrote the paper. All authors read and approved the paper.

## Supplementary Material

Additional file 1 **Supplement Diversity Graph construction**. Supplemental material showing analytically whether the construction of the diversity graph depends on the initial amount of the main source in a batch culture. Click here for file

Additional file 2 **Supplement Structural Analysis**. Supplemental material providing further information regarding the definition and application of graph-theoretic measures on the diversity graphs. Click here for file

Additional file 3 **Supplement Functional Analysis**. Supplemental material including additional information on the simulations of strain communities in batch cultures under several growth conditions. Click here for file
