# Supplementary material for: A computational exploration of bacterial metabolic diversity identifying metabolic interactions and growth-efficient strain communities
Source: BMC Syst Biol. 2011 Oct 18;5:167. doi: 10.1186/1752-0509-5-167 (PMC3212978; doi:10.1186/1752-0509-5-167)
Supplement: Additional file 1 — Supplement Diversity Graph construction. Supplemental material showing analytically whether the construction of the diversity graph depends on the initial amount of the main source in a batch culture. [file 1752-0509-5-167-S1.PDF]

Additional file 1: Supplement Diversity Graph construction (DG)

The question here is whether the construction of the diversity graph depends on the initial amount of the main source in a batch culture or remains robust when this quantity changes. The robustness of the structure can be determined by the way the edge weights are actually affected under changes in the initial concentration of the main source.

The concentration value of an exchange molecule at a time t depends on the uptake for consumption (or intake for production) rate u of the specific molecule, the growth rate μ, the initial biomass b_0 and the initial concentration value C_{t_0} of the molecule according to equation [2]. A byproduct p reaches its maximum concentration value at the time t_{exh} (equation [4]) where the main source m is getting exhausted (equation [3]).

Under the hypothesis that during the growth phase that correspond to the metabolism of the main source, the growth rate and the uptake (or intake) rate remain constant the amount of the produced nutrient is proportional to the initial amount of the main source (equation [5]). The hypothesis of constant flux rates is actually true as long as sufficiently small time intervals can be considered. In that case, the relative concentration difference of a byproduct that determines the edge weight according to equation [1] is proved to be independent on the initial amount of the main source. Therefore, the diversity graph representation remains unaffected under changes in the initial amount of the main source.

$$\text{edge weight}: w_{ij} = \max_s\left(\frac{\left|{}_{\max}C_i^s - {}_{\max}C_j^s\right|}{\max({}_{\max}C_i^s, {}_{\max}C_j^s)}\right), \quad \text{where } {}_{\max}C_i^s = \max\left(C_i^s(t)\right) \quad [1]$$

$$C_t = C_{t_0} - \frac{u}{\mu} b_0 (1 - e^{\mu t}) \quad [2]$$

Main source:

$$C_{t_{exh}}^m = 0,\ [1] \Rightarrow C_{t_0}^m = \frac{u^m}{\mu} b_0 (1 - e^{\mu t_{exh}}) \quad [3]$$

By-production:

$$C_{t_0}^p = 0,\ [1] \Rightarrow C_{t_{exh}}^p = \frac{u^p}{\mu} b_0 (1 - e^{\mu t_{exh}}) \quad [4]$$

$$[3],[4] \Rightarrow C_{t_{exh}}^p = \frac{u^p}{u^m} C_{t_0}^m \quad [5]$$
