# Supplementary material for: A computational exploration of bacterial metabolic diversity identifying metabolic interactions and growth-efficient strain communities
Source: BMC Syst Biol. 2011 Oct 18;5:167. doi: 10.1186/1752-0509-5-167 (PMC3212978; doi:10.1186/1752-0509-5-167)
Supplement: Additional file 2 — Supplement Structural Analysis. Supplemental material providing further information regarding the definition and application of graph-theoretic measures on the diversity graphs. [file 1752-0509-5-167-S2.PDF]

Additional file 2: Supplement Structural Analysis (SA)

This file includes additional information regarding the graph-theoretic measures that are applied on diversity graphs. Diversity graphs are constructed in order to represent pair-wise differences in by-production between *E. coli* strains (see Methods in the main text). Each single-carbon-source growth condition corresponds to a different diversity graph. The diversity graphs are weighted (undirected) graphs, in which weights take values between 0 and 1.

The edge weight distribution is shown in Figure 1 for each of the diversity graphs and in Figure 2 for a few representative examples.

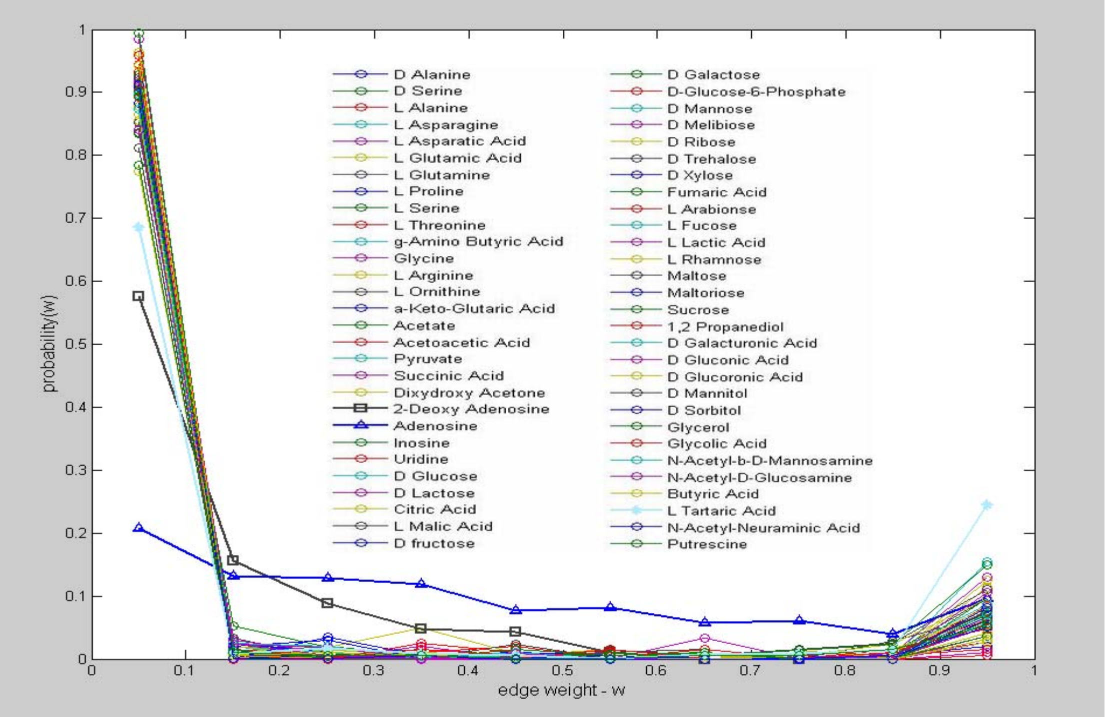

Figure 1 Edge weight distribution of each diversity graph. In most of the single-carbon-source conditions, the majority of edges in the corresponding diversity graphs have low weight values. Exceptions are growth on *Adenosine* (blue line with triangular markers) and *2-Deoxy Adenosine* (black line with square markers).

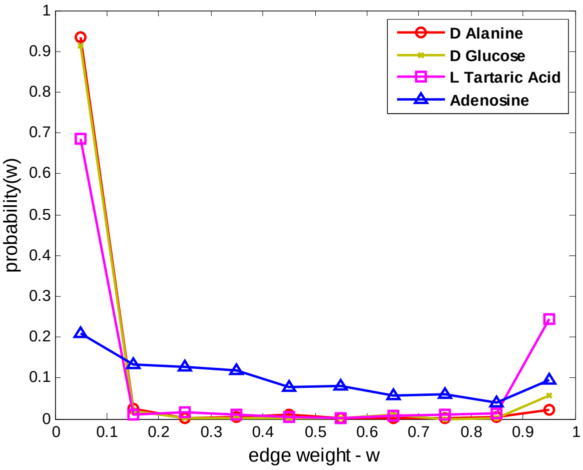

Figure 2 Edge weight distribution of a few representative diversity graphs. As shown in Fig. 1, most edge weight distributions are similar to the distributions of *D Alanine* and *D Glucose*. *Adenosine* is an exception.

1. Degree and Strength centrality

Definition

In a binary graph, in which edges are either present or absent, the most fundamental feature of a node in a graph is its *degree*. The node degree is equivalent to the simplest centrality measure known as *degree centrality*. The degree of a node v, written $d(v)$, is defined as the number of edges incident with v. The degree is a local measure, which expresses the importance of a node in a graph with respect to its connections.

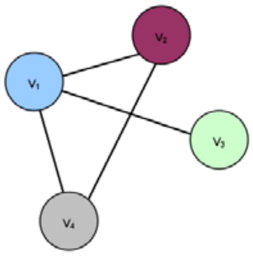

Figure 3 A simple graph consisting of 4 nodes and 4 edges. The degree of each node is: $d(v_1)=3, d(v_2)=2, d(v_3)=1, d(v_4)=2$

The degree has been extended to take into account the weight values of the edges when analyzing weighted graphs [1, 2]. The *strength* of a node v, also known as *strength centrality,* $s(v)$, is defined as the sum of the weights of the edges incident with the node (Equation 1). The normalized strength of a node v, $C_s(v)$ corresponds to the mean value of the edge weights w of its neighbors (Equation 2). This definition is also applicable to the binary graph representations, in which edges are either present or absent so that they can be considered to take weight values either 1 or 0 respectively. In that case, the node strength corresponds to the node degree. In grayscale networks [3], the normalized strength centrality ranges from 0 to 1, where 0 implies a disconnected node and 1 a node connected with all other nodes of the graph, with weights equal to 1.

$$s(v) = \sum_{e=\{v,u\}\in E} w(e) \qquad (1)$$

$$C_s(v) = \frac{1}{n-1} s(v) \qquad (2)$$

The *degree distribution,* $P(k)$, expresses the probability that a selected node v has degree $d(v)$ equal to k. The probability $P(k)$ is estimated by counting the number of nodes $N(k)$ having degree k and dividing by the total number of nodes n. Similarly for weighted graphs, the *strength distribution* $P(s)$ corresponds to the probability that a selected node has strength s.

Let v be the node that exhibits the highest centrality over all nodes in the graph, noted as $C_x^*(v)$. The *network centrality* $C_x(G)$ of a graph G is then determined by the mean value of the differences between the maximum centrality $C_x^*(v)$ and the centrality of each node in the graph, where the index x represents whether the node centrality is measured with respect to strength or degree (Equation 3).

$$C_x(G) = \frac{1}{n-2} \sum_{u\in V} \left(C_x^*(v) - C_x(u) \right) \qquad (3)$$

The network centrality takes values within [0, 1]. A star topology, in which at most one node has degree greater than one, has network centrality equal to 1. A complete graph, on the other hand, in which all nodes have the same degree, has network centrality equal to 0.

Centrality in Diversity graphs

Table SA 1: Network Centrality over different carbon sources

**WEIGHTED GRAPHS**	**mean Centrality**	**std Centrality**	**min-max Centrality**	**Carbon of min Centrality**	**Carbon of max Centrality**
	0.9165	0.0637	0.6147 – 1	'adenosine'	'acetate'

**BINARY GRAPHS**	**mean Centrality**	**std Centrality**	**min-max Centrality**	**Carbon of min Centrality**	**Carbon of max Centrality**
	0.9289	0.0543	0.7297 – 1	'L Tartaric Acid'	'acetate', 'glycolate'

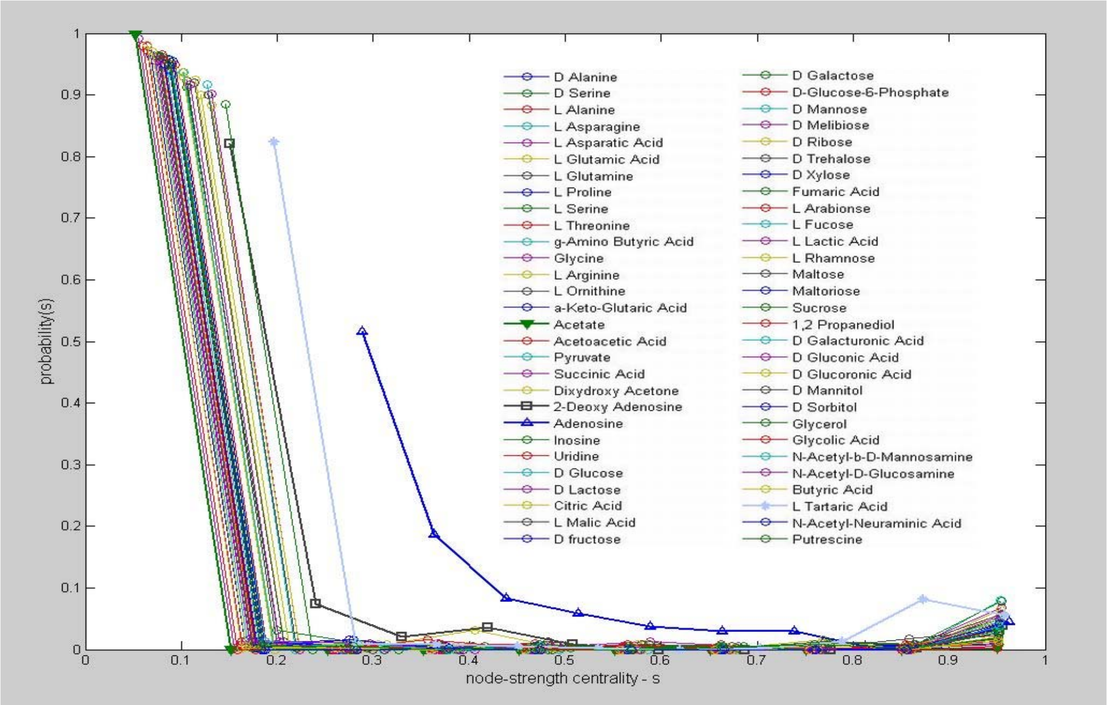

Figure 4 Node-strength centrality distribution of each different carbon-source diversity graph. Most nodes exhibit low centrality (less than 0.2), whereas a few nodes have considerably high strength centrality (above 0.9). The diversity graph of *adenosine* exhibits a broader strength centrality distribution than the rest graphs.

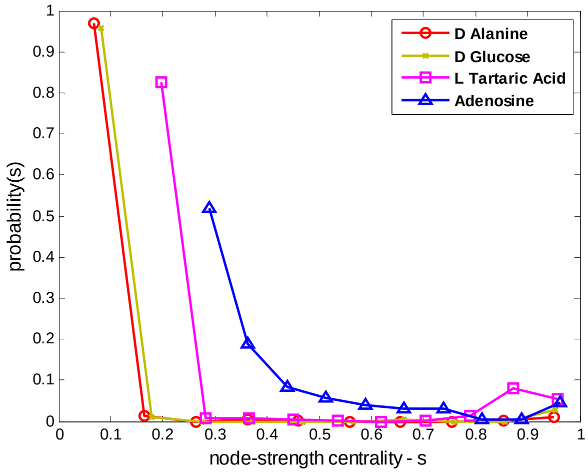

Figure 5 Node-strength centrality distribution of a few representative diversity graphs.

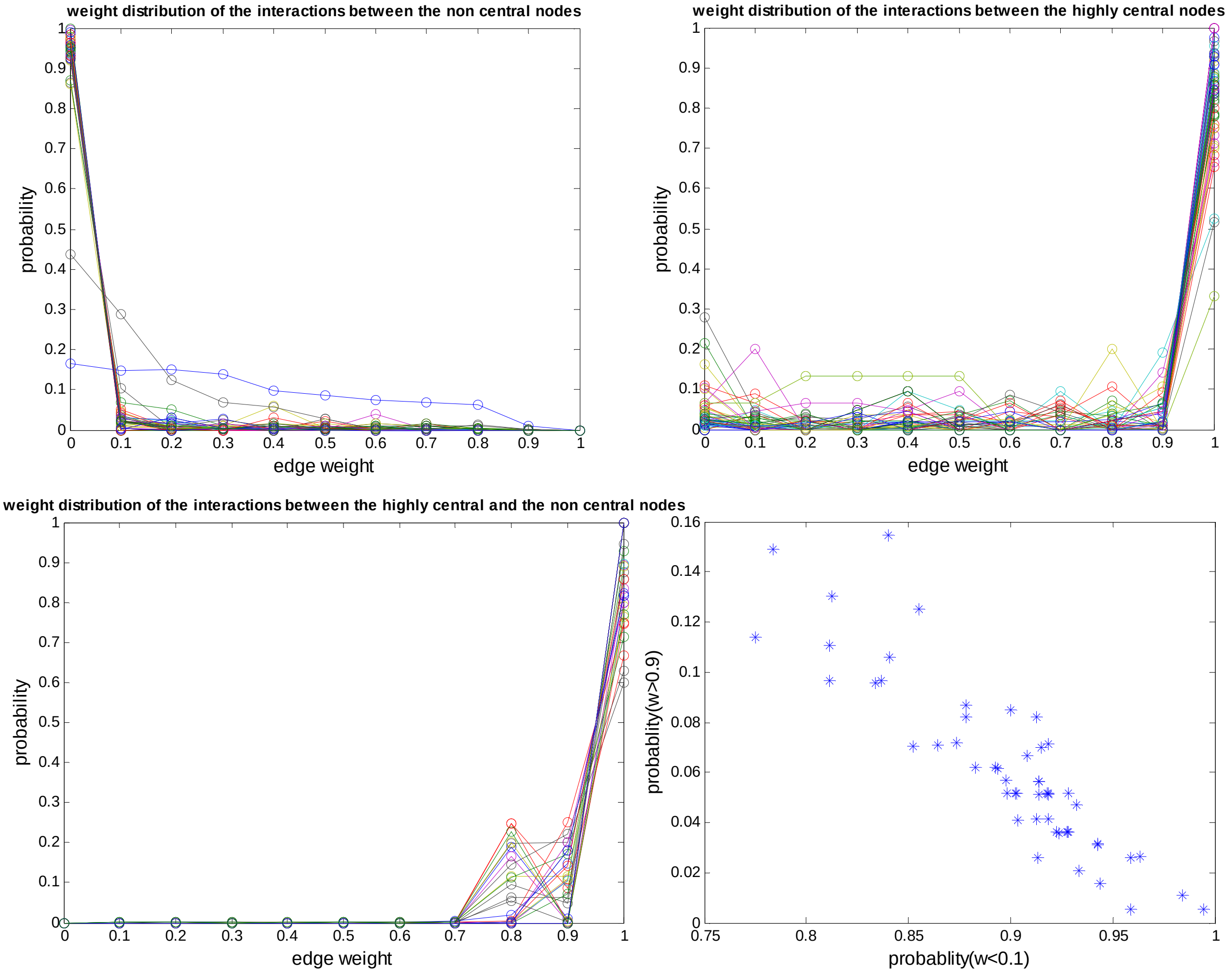

Figure 6 (Up-Left) The edge weight distributions of the connections among the non-central nodes of the diversity graph of each growth condition. Most edges exhibit weight values less than 0.1, which indicates that the non-central nodes are metabolically similar with each other. (Up-Right) The edge weight distributions of the connections among the highly central nodes of the diversity graph of each growth condition. The central nodes are mostly highly connected with each other. (Bottom-Left) The edge weight distributions of the connections between the highly central nodes and the non-central nodes of the diversity graph of each growth condition. The non-central nodes are strongly connected with the central nodes. (Bottom-Right) The percentage of the strong interactions in the graph linearly depends on the percentage of the low weighted edges (R=-0.8951, p-value $< 10^{-20}$) verifying the interdependence between the central and the non-central nodes of the graph.

2. Maximum cliques

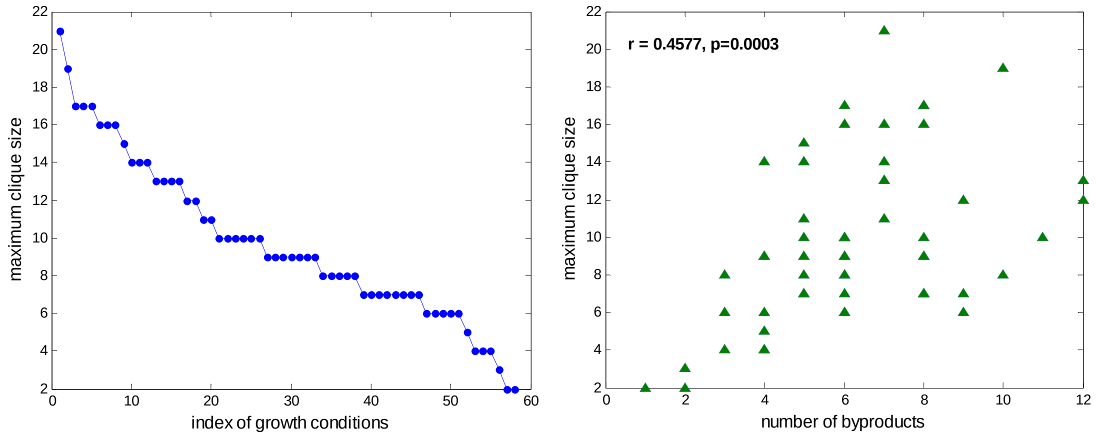

Figure 7 (Left) The maximum clique size as found in the binary representation of the diversity graph of each growth condition. (Right) The number of the metabolites that are by-produced in each growth condition with respect to the maximum clique size.

3. Assortativity coefficient

Definition

The assortativity coefficient has been proposed [3-5] to quantify the degree of similarity (or dissimilarity) of the degrees (or other features that can characterize a node such as age, race, cultural trait, evolutionary trait) of adjacent nodes in a graph, thus reflecting the preference for nodes to attach to other nodes in a network that are similar (or dissimilar) in some way. Specifically, the assortativity coefficient corresponds to the Pearson product-moment correlation coefficient of the features of adjacent nodes of a graph and lies in the range [-1, 1], where -1 expresses perfect disassortative networks and 1 indicates perfect assortativity. The assortativity coefficient was initially proposed by Newman [4] and was applied in several network models and real networks in order to study their assortative mixing by degree. The generalization of the assortative coefficient for weighted graphs has also been proposed [5] to reflect the tendency of having a high-weighted link between two nodes of similar degrees (or other features).

For any scalar property $\rho : V \to \Re$ assigned to the nodes of the graph, the weighted clustering coefficient r^w is defined as follows [3]:

$$r^w = \frac{4H \sum_{\{u,v\}\in E} w(u,v)\rho(u)\rho(v) - \left[\sum_{\{u,v\}\in E} w(u,v)(\rho(u)+\rho(v))\right]^2}{2H \sum_{\{u,v\}\in E} w(u,v)(\rho(u)^2+\rho(v)^2) - \left[\sum_{\{u,v\}\in E} w(u,v)(\rho(u)+\rho(v))\right]^2} \qquad (4)$$

The term $w(u,v)$ corresponds to the weight value of the edge (u,v) and H is the total weight of all edges of the graph. If all the edge weights of the graph are equal then the weighted clustering coefficient reduces to the coefficient as defined for the binary graphs.

Assortativity by ERI in Diversity graphs

The assortativity coefficient is investigated with respect to the Evolutionary Retention Index (ERI) [6] of the deleted gene that characterizes the mutant-node. The higher the conservation value of the gene under deletion is across divergent bacteria, the lower becomes the probability of the specific mutant to evolve. A disassortative mixing is observed for most carbon conditions as shown in Table SA 2 indicating a tendency of potential cross-feeding interactions to be developed between mutants derived from the deletion of a highly conserved gene and mutants derived from the deletion of a less conserved gene. This observation is important since it suggests that when the cell loses an essential, non-lethal, highly conserved gene then it can metabolically interact with another mutant who is not unlikely to evolve in a population and get rescued from extinction.

Table SA 2: Assortativity Coefficient (by ERI) over different carbon sources

WEIGHTED GRAPHS	mean Assortativity	std Assortativity	min-max Assortativity	Carbon of min Assortativity	Carbon of max Assortativity
	-0.2251	0.1117	-0.4431 - -0.0064	'acetoacetic acid'	'adenosine'

BINARY GRAPHS	mean Assortativity	std Assortativity	min-max Assortativity	Carbon of min Assortativity	Carbon of max Assortativity
	-0.2365	0.1219	-0.5014 - -0.0097	'dixydroxy acetone'	'adenosine'

Assortativity by strength in Diversity graphs

When the centrality of each node in the network is taken as a node property, the assortativity coefficient is observed to take a negative value close to -1 for the most of the growth conditions showing that the diversity graphs are highly disassortative. The observed disassortative mixing is a direct consequence of the structure of the diversity graphs where (few) highly central nodes are connected with the (many) non-central nodes. Table 5.5 shows the mean, the standard deviation as well as the minimum and maximum assortativity values over all graphs. Among all growth conditions, less disassortative is the diversity graph, which corresponds to the carbon source *adenosine*. The binary diversity graphs of *acetate* and *glycolate* have assortativity coefficient equal to -1, which is a direct consequence of their star topology. Since the binary representation strengthens the divergence between the central and the non-central nodes the assortativity coefficient is observed to be affected towards higher assortativity values.

Table SA 3: Assortativity Coefficient (by strength) over different carbon sources

WEIGHTED GRAPHS	mean Assortativity	std Assortativity	min-max Assortativity	Carbon of min Assortativity	Carbon of max Assortativity
	-0.7868	0.110	-1 – -0.3041	'acetate'	'adenosine'

BINARY GRAPHS	mean Assortativity	std Assortativity	min-max Assortativity	Carbon of min Assortativity	Carbon of max Assortativity
	-0.9638	0.0399	-1 – -0.7212	'acetate', 'glycolate'	'adenosine'

4. Clustering coefficient

Definition

The clustering coefficient was first proposed by Watts and Strogatz [7] as a measure of the cliquishness of a neighborhood. Specifically, the clustering coefficient, C_v,

ranges from 0 to 1 and expresses the probability of two adjacent nodes to a reference node v to also have a direct link. The clustering coefficient has been extended for weighted undirected graphs. The definition, which is shown in Equation 5has been introduced in [8] and can be applied in both binary and weighted representations of networks of weight values within (0, 1]:

$$C_v = \frac{\sum_{i=1}^{n}\sum_{j=1}^{n} w_{vi} w_{vj} w_{ij}}{\sum_{i=1}^{n}\sum_{j=1, i\neq j}^{n} w_{vi} w_{vj}} \qquad (5)$$

$$C(G) = \sum_{v \in V} C_v / n \qquad (6)$$

The network clustering coefficient $C(G)$ of a graph G, which is given by the average of the clustering coefficients (Equation 6), measures the global density of interconnected triplets in the graph and expresses the overall tendency of nodes to participate in clusters.

Clustering coefficient in Diversity graphs

As shown in Table SA 4, the diversity graph of *glycine* shows the highest value of the clustering coefficient. The diversity graphs of the *acetate* and *glycolate* on the other hand, have clustering coefficient equal to 0 or very close to 0, in all their diversity graph definitions, which is consistent with their start-like topology that does not allow other connections but those with the single central node. The diversity graphs, as mentioned previously, are highly centralized, consisting of few highly central nodes that are actually connected to many highly non-central nodes. Given this structural information, the high values of the network clustering coefficient imply that the central nodes are highly connected with each other forming a highly clustered area in the graph. The exact dependence between clustering coefficient and centrality is shown in figure SA 5. The clustering coefficient exhibits a non-linear dependence on the strength centrality. Highly non-central nodes (low strength centrality) exhibit clustering coefficient, which is considerably high (close to one). The central nodes are connected with a loosely connected area of relatively high size, which corresponds to the redundant group of mutants. Thus, the redundant group is part of the highly clustered areas and therefore their clustering coefficient is high. The mean clustering coefficient of the network remains however at a high level (Table 5.7) due to the fact that most of the nodes of the graph have low strength centrality.

Table SA 4: Network Clustering Coefficient over different carbon sources

WEIGHTED GRAPHS	mean Clustering	std Clustering	min-max Clustering	Carbon of min Clustering	Carbon of max Clustering
	0.8207	0.1449	0 – 0.9695	‘acetate’	‘glycine’

BINARY GRAPHS	mean Clustering	std Clustering	min-max Clustering	Carbon of min Clustering	Carbon of max Clustering
	0.8126	0.2023	0 – 0.9946	‘acetate’, ‘glycolate’	‘glycine’

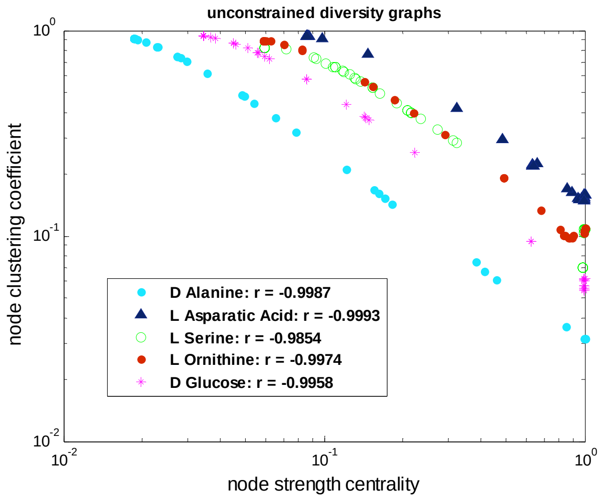

Figure 8 The node's clustering coefficient with respect to the strength centrality for five different growth conditions approximates a straight line on a log-log plot. The correlation coefficient values – r are shown (p-value = 0 in all cases). Highly central nodes have low clustering coefficient whereas the nodes of significantly low strength centrality appear to have high correlation coefficient value.

4. By-products across carbon-conditions

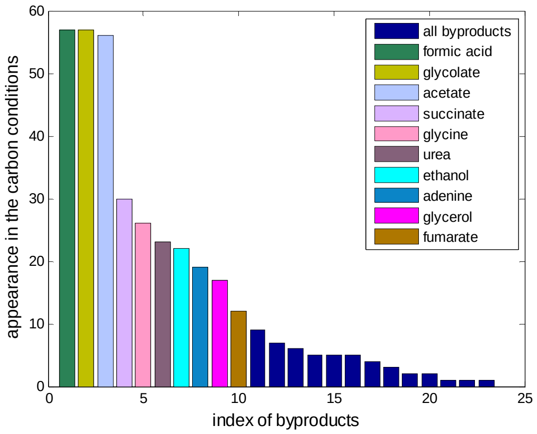

Figure 9 23 different metabolites were observed as by-products across the different carbon conditions under the applied genetic perturbations. The number of the growth conditions where each of these byproducts is present is shown. The most frequently-appearing metabolites are highlighted.

References

1. Barrat A, Barthelemy M, Pastor-Satorras R, Vespignani A: The architecture of complex weighted networks. *Proceedings of the National Academy of Sciences of the United States of America* 2004, 101(11):3747-3752.
2. Newman ME: Analysis of weighted networks. *Physical review* 2004, 70(5 Pt 2):056131.
3. Tsiaras LV: Algorithms for the analysis and visualization of biomedical networks. *PhD Thesis* 2009 (Computer science department, University of Crete, Greece).
4. Newman ME: Assortativity mixing in networks. *Phys Rev Lett* 2002, 89(20):208701.
5. Leung CC, Chau HF: Weighted assortative and disassortative networks model. *Physica A: Statistical Mechanics and its Applications* 2007, 378(2):591-602.
6. Gerdes SY, Scholle MD, Campbell JW, Balazsi G, Ravasz E, Daugherty MD, Somera AL, Kyrpides NC, Anderson I, Gelfand MS *et al*: Experimental determination and system level analysis of essential genes in Escherichia coli MG1655. *Journal of bacteriology* 2003, 185(19):5673-5684.
7. Watts DJ, Strogatz SH: Collective dynamics of 'small-world' networks. *Nature* 1998, 393(6684):440-442.
8. Kalna G, Higham JD: A clustering coefficient for weighted networks, with application to gene expression data. *AI Communications* 2007, 20:263-271.
