# Supplementary material for: A computational exploration of bacterial metabolic diversity identifying metabolic interactions and growth-efficient strain communities
Source: BMC Syst Biol. 2011 Oct 18;5:167. doi: 10.1186/1752-0509-5-167 (PMC3212978; doi:10.1186/1752-0509-5-167)
Supplement: Additional file 3 — Supplement Functional Analysis. Supplemental material including additional information on the simulations of strain communities in batch cultures under several growth conditions. [file 1752-0509-5-167-S3.PDF]

Additional file 3: Supplement Functional Analysis (FA)

This file includes additional information regarding the simulations of strain communities in batch cultures under several growth conditions. The strain communities correspond to cliques in the diversity graphs. The cliques are identified using the binary representation of the diversity graph. In this work, the binary representations are produced using a threshold of 0.6. The functional analysis of the diversity graphs aims to identify beneficial strain communities performing better than their corresponding monocultures and reveal the extent of the metabolites that are exchanged. We further explore whether the growth of strain communities depends on the growth of their constituent parts.

Benefit

The growth performance corresponds to the endpoint biomass concentration of a population in a specific growth condition. Two definitions are given, the *absolute* and the *relative* benefit. If the group performance g of the heterogeneous cell population is superior to the performance of any (wild type or mutant) monoculture, m_i, then the heterogeneous community under study is beneficial. We term this benefit 'absolute'. The condition of 'any' can be relaxed, so that the growth performance of the heterogeneous population is compared to the homogeneous performances of all the members which constitute the community under study. In this case we call the benefit 'relative'.

$$\text{benefit} = \frac{(g - \max_i(m_i))}{\max_i(m_i)} \qquad (1)$$

Predictability of Growth performance

It is worthwhile to explore whether the growth of strain communities depends on the growth of their constituent parts. Here, we specifically explore the dependence of the growth of a community on its constituent pairs (hypothesis). Given a specific single-carbon-source batch scenario, all strain communities are simulated as well as all their constituent strain pairs. A clique of size k represents a community of k strains in the diversity graph and contains $k \cdot (k-1)/2$ edges that correspond to its constituent strain pairs (Figure 1). We assign to each different strain pair a new weight (different that what is given in the diversity graph), which corresponds to its simulated growth performance in co-culture and we explore how the mean of the growth performances of the pairs (prediction) is related to the simulated growth performance of the community (simulation).

Interestingly, throughout this analysis for several different growth conditions we observed that the growth performance of a strain community of any size linearly depends on the mean of the growth performances of its constituent strain pairs as long as each of these pairs is not unexploited regarding by-production. A strain community is considered unexploited if cross-feeding interactions either do not occur or do not fully exploit the metabolites produced in co-culture. However, unexploited mutant pairs exist and play an important, novel role in larger communities.

Effective weights

In a similar manner, we further explore the dependence of the growth of a community on its constituent triplets. Thus, in order to validate the extent of the growth predictability (hypothesis), we introduce the concept of effective weights between the strain-pairs (Figure

1). Specifically, instead of using the weights, which corresponds to the simulated growth performance of the pairs as before, we now focus on the unexploited strain pairs and give them an effective weight, which is estimated by the difference between the simulated performance of the triplet and its prediction calculated as before by the mean of the performances of its pairs (Figure 1). Consistent divergence between the simulated performance of the triplets and the predicted performance calculated by their constituent pairs is observed as groups of lines and not as the presence of a few outliers and corresponds to edges between highly central nodes because these edges consistently participate in the formation of many different communities. For this reason, effective weights are only assigned to edges that connect highly central nodes with each other.

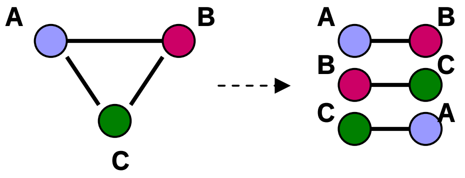

Figure 1 Let $P_{sim}(A,B,C)$ be the simulated growth performance of the triplet consisting of the strains A, B and C and $P_{sim}(A,B)$, $P_{sim}(B,C)$ and $P_{sim}(A,C)$ be the simulated growth performances of the corresponding strain pairs. Let $P_{pred}(A,B,C)$ be the predicted performance of the specific triplet, which is estimated as follows (hypothesis): $P_{pred}(A,B,C) = \frac{P_{sim}(A,B) + P_{sim}(B,C) + P_{sim}(A,C)}{3}$. The relation between $P_{sim}(A,B,C)$ and $P_{pred}(A,B,C)$ is linear as long as unexploited strain pairs are not present. Let the pair (A,B) be an unexploited pair, then we give it an effective weight w_{eff} , which is defined by the difference $w_{eff} = P_{sim}(A,B,C) - P_{pred}(A,B,C)$. By assigning effective weights to specific (unexploited) strain pairs, we explore in a similar manner the dependence of the growth of larger communities (than triplets) on their constituent (now differently weighted) pairs. This dependence is linear for the growth conditions tested.

1. Growth on *Glycolate*

Single-growth characteristics

Growth on *glycolate* produces a diversity graph of star topology with only one mutant - the mutant of the deleted gene 'b2276'- to be different from the rest mutants of the graph (including the wild-type cell). Because of this topology, the potential bacterial communities exclusively consist of mutant pairs of which the mutant of 'b2276' is omnipresent. This particular mutant is the only mutant capable of by-producing *acetate* and the only one incapable of producing *formic acid* contrary to the rest mutants (**Fig. 1.1**). *Acetate* and *formic acid* are actually the two metabolites that are observed to be produced by the system. The gene 'b2276' exhibits unique metabolic properties in other growth conditions as well. When deleted the cell exhibits poor growth performance (~20% reduction) compared to the growth performance of the wild-type cell in the same initial growth conditions. From all single-gene knockout mutants simulated to grow independently on limited *glycolate* no mutant is observed to be capable of combining maximum ability to metabolize *glycolate* with maximum ability to metabolize *acetate*, which is an essential by-product for growth. Furthermore, no mutant is observed to perform better than the wild-type cell (**Fig. 1.1**).

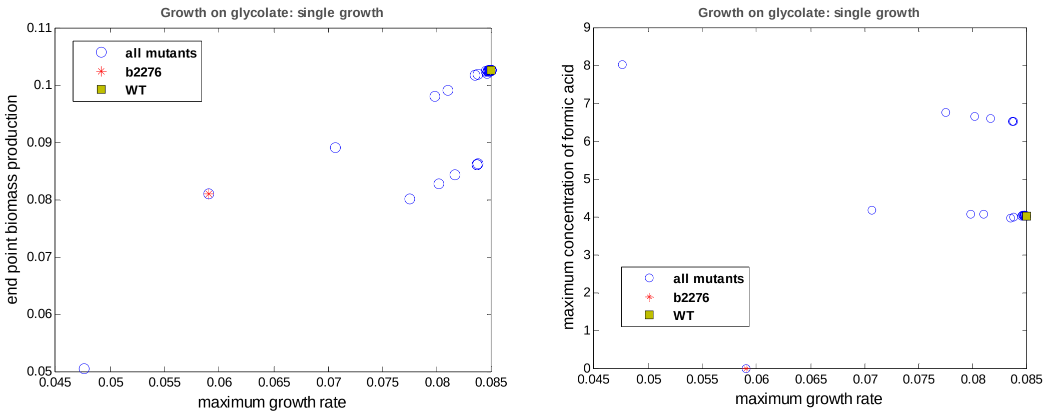

Figure 1.1 (Left) The maximum growth rate (h^{-1}) with respect to the end-point biomass concentration (gDW/L) for each viable mutant growing independently on limited *glycolate*. (Right) All the mutants apart from the 'b2276' produce *formic acid*. The maximum growth rate with respect to the maximum concentration of *formic acid* is shown. The wild-type cell and the mutant 'b2276' are highlighted.

Two-competitor growth

The coexistence of the mutant of the deleted gene 'b2276' with any other mutant is observed to be beneficial, which means that the endpoint biomass concentration of the group is greater than the endpoint biomass concentration of each involved mutant growing independently as a monoculture (positive relative benefit). Absolute benefit is observed as well, which indicates that there are mutant coexistences able to better exploit the given environment than any single-gene knocked out mutant growing independently in the same initial conditions. The mutant of the deleted gene 'b2276' seems to have an altruistic behavior in its communities because of the *acetate* it provides to the growth medium. Nevertheless, it is observed to exploit the *formic acid* that is provided in the growth medium by the partner mutant increasing its growth rate (**Fig. 1.2**).

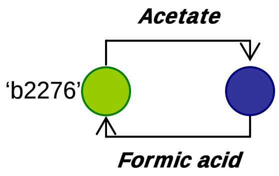

Figure 1.2 A cartoon diagram of the cross-feeding interactions, which occur when the mutant 'b2276' and any other mutant (or the wild-type) co-grow on limited *glycolate.* Their coexistence is beneficial.

A metabolically interacting mutant pair consisting of the mutants derived from the deletion of the gene ‘b2276’ and the gene ‘b3708’ respectively is analytically described in the following. The flux rate time profiles (**Fig. 1.3**) verifies the exchange of the two products *acetate* and *formic acid* between them. Each novel metabolite is consumed simultaneously with the primal source. It is also observed that the growth rate of the mutant of ‘b2276’ is increased by the presence of *formic acid* in the growth medium and that also the growth rate of the mutant of ‘b3708’ is beneficially affected by the presence of the novel metabolite *acetate* in the growth medium (**Fig. 1.4**).

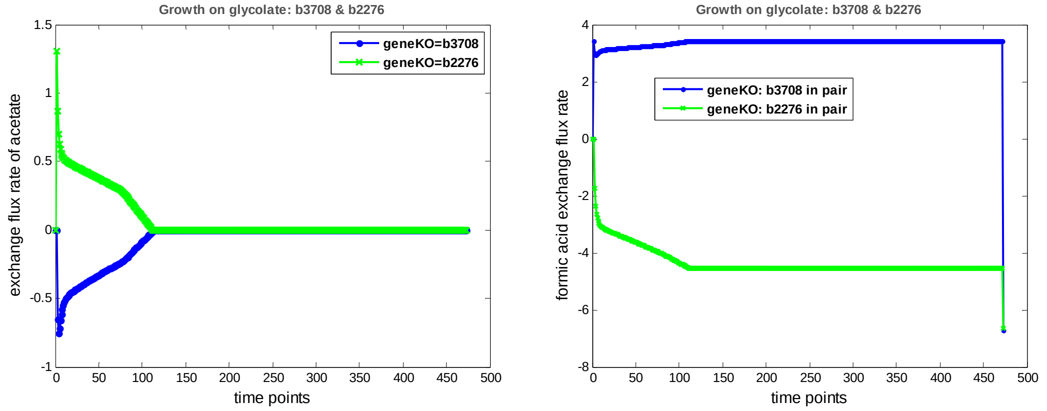

Figure 1.3 Two mutants, the ‘b2276’ and the ‘b3708’, co-grow on limited *glycolate.* The flux rate (mmol gDW^{-1} h^{-1}) time profiles of two metabolites *acetate* (Left) and *formic acid* (Right) are shown for each mutant under competition. Each time point corresponds to 0.1 h.

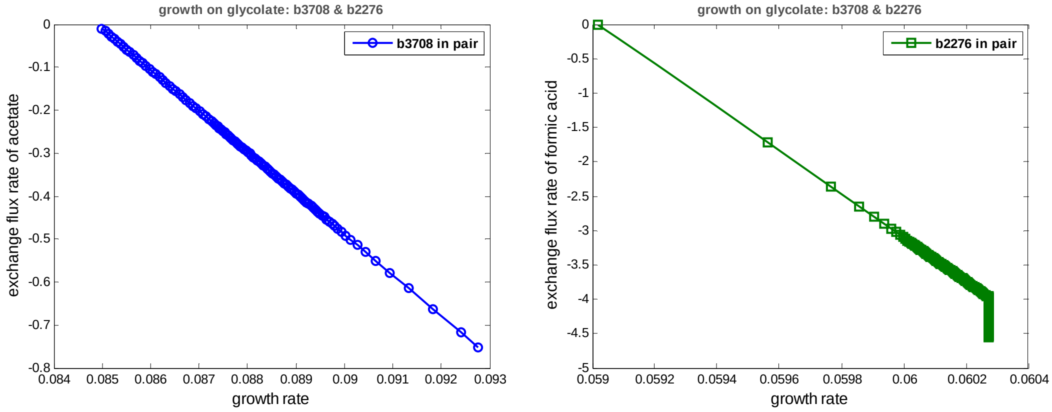

Figure 1.4 (Left) The growth rate of the mutant ‘b3708’ is increased as the concentration of *acetate* increases in the medium and consequently its uptake flux. (Right) The growth rate of the mutant ‘b2276’ is increased as the concentration of *formic acid* increases in the common medium and consequently its uptake flux.

2. Growth on *Pyruvate*

Single-growth characteristics

The set of the metabolites that are observed to be by-produced by the mutants during their independent growth on limited *pyruvate* include: *acetate*, *formic acid*, *glycine* and *glycolate.* The maximum clique size that exists in the diversity graph of *pyruvate* is 6, whereas the number of the highly central nodes that correspond to mutants of highly different metabolic capabilities with respect to by-production is 7. Among all single-gene knockout mutants, the mutant derived from the deletion of the gene b3403 is observed to exhibit the maximum growth performance, which is 4.7% increased with respect to the performance of the wild-type cell population (**Fig. 2.1**). The gene b3403 is not highly conserved among different bacterial species (ERI equals to 0.38), encodes the *phosphoenolpyruvate carboxykinase* and participates in the *anaplerotic* reactions of the *E. coli* metabolism. The underlying reason for its high growth performance is the efficient strategy which follows during its growth even if it costs to the cell a lower growth rate with respect to the wild type during the metabolism of *pyruvate*. Specifically, this mutant exhibits a relatively high by-production of *acetate*, which uses as secondary resource when *pyruvate* is exhausted (**Fig. 2.1**).

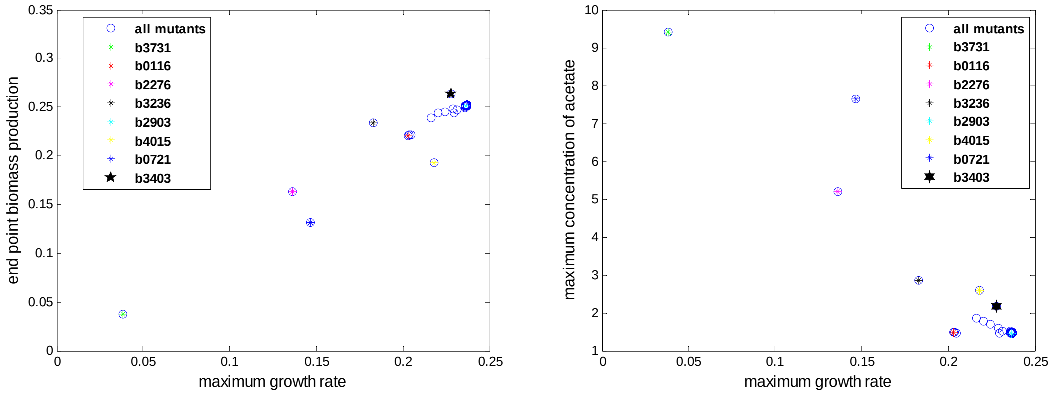

Figure 2.1 (Left) The maximum growth rate with respect to the end-point biomass concentration for each viable mutant growing independently on limited *pyruvate*. (Right) The maximum growth rate with respect to the maximum concentration of *acetate* is shown. The highly central mutants are highlighted. The most efficient single-growth mutant (b3403) is also depicted.

Two-competitor growth

The absolute and relative benefits of all the examined mutant pairs when co-grow on limited *pyruvate* are shown sorted in **Figure 2.2**. The highly central mutant involved in each pair is also shown. It can be observed that the mutant b3236 and b0116 exhibit consistently negative absolute benefit in any pair, whereas the mutant b3236 exhibits in addition consistent negative relative benefit indicating its inefficient contribution in co-growth.

On the other hand, when the environmental-invariant mutant of the deleted gene b3731 coexists with the mutant of the deleted gene b3403, which exhibits the best single growth performance on *pyruvate*, they beneficially interact (absolute and relative benefit equals to 0.8%). The mutant of the deleted gene b3731 exhibits the lowest growth rate of all the viable mutants (**Fig. 2.1**) that can be derived from single-gene knockouts that grow on limited *pyruvate*. This mutant is also observed to exhibit the maximum *acetate* production (**Fig. 2.1**). However, it is not capable of consuming the *acetate* it produces. The interaction concerns the exchange of *acetate* and *formic acid* from the b3731 to the b3403 because the last is the only mutant that is capable of consuming these metabolites. Furthermore, the interaction concerns the exchange of *glycolate* from the mutant of b3403 to the b3731 as can be seen in the exchange flux rate profiles (**Fig. 2.3**). It is observed that the presence of

glycolate increases the growth rate of the mutant of b3731. The growth rate depends linearly on the uptake flux rate of *glycolate* (**Fig. 2.3**). The absolute value of the uptake rate increases as the amount of *glycolate* increases in the growth medium since the availability of the metabolite directly shapes the corresponding capacity flux bounds.

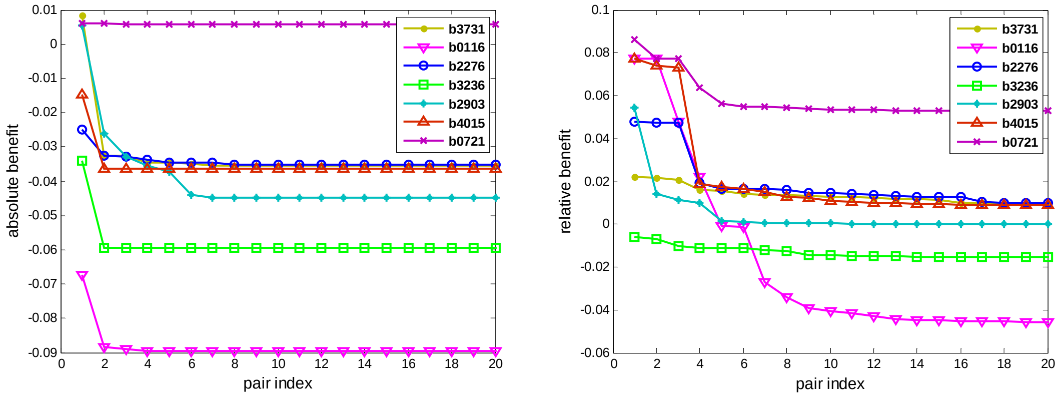

Figure 2.2 Each mutant pair corresponds to the potential interaction between a highly central mutant and another mutant in the diversity graph. (Left) The absolute benefit of the mutant pairs when co-grow on limited *pyruvate*. (Right) The relative benefit of the mutant pairs.

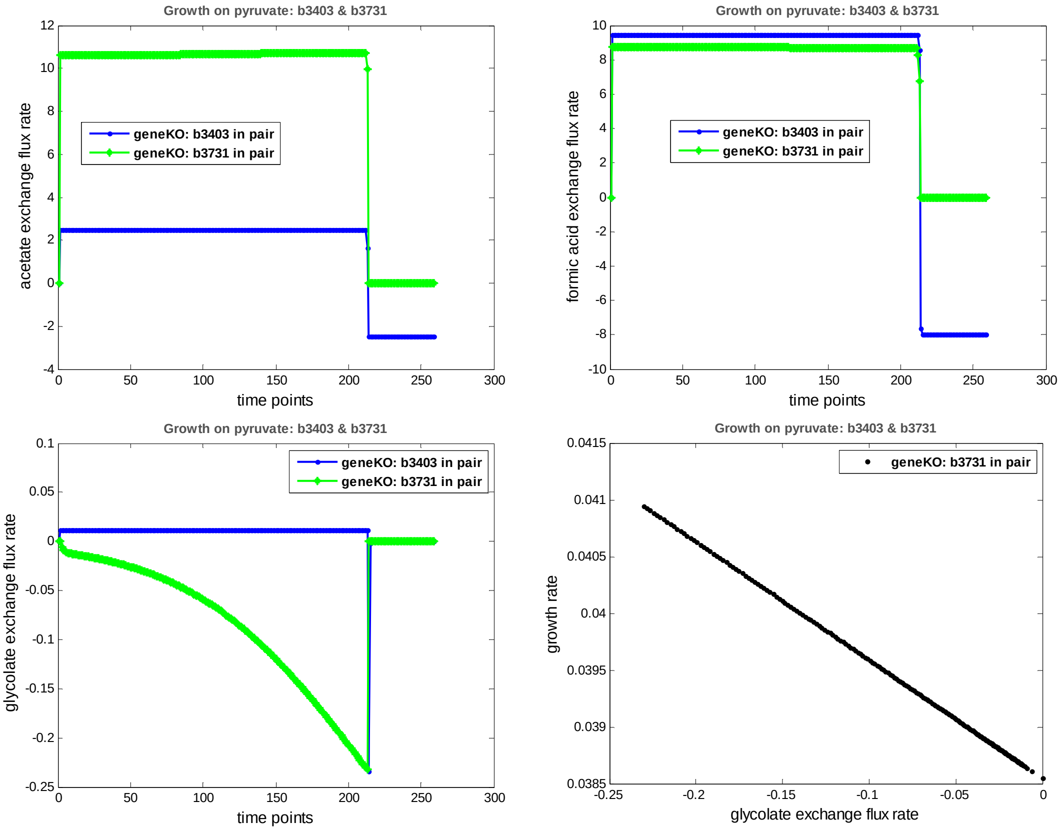

Figure 2.3 Two mutants, the b3403 and the b3731, co-grow on limited *pyruvate.* The flux rate time profiles of the metabolites *acetate* (Top-Left), *formic acid* (Top-Right) and *glycolate* (Bottom-Left) are shown for each mutant under competition. The growth rate of the mutant b3731 is increased as the concentration of *glycolate* increases in the medium and consequently its uptake flux.

The growth performance of the most beneficial mutant pair (among all the pairs) has been also explored for different initial ratios of the mutants in the population. It is observed (**Fig. 2.4-Right**) that maximum growth benefit equals to 0.8% is achieved when the two mutants

are initially in equal frequency in the population. On the other hand, another pair, which consists of the mutants of the deleted gene b3403 and b0721 respectively, is observed to exhibit beneficial growth performance of absolute benefit equal to 1.1% at the initial ratio of 0.9:0.1 of b3403:b0721 (**Fig. 2.4–Left**). This example is presented in order to underline the importance of the initial population ratios to the group performance of the communities and that exploring equal initial frequencies covers a sub-space of potential beneficial communities.

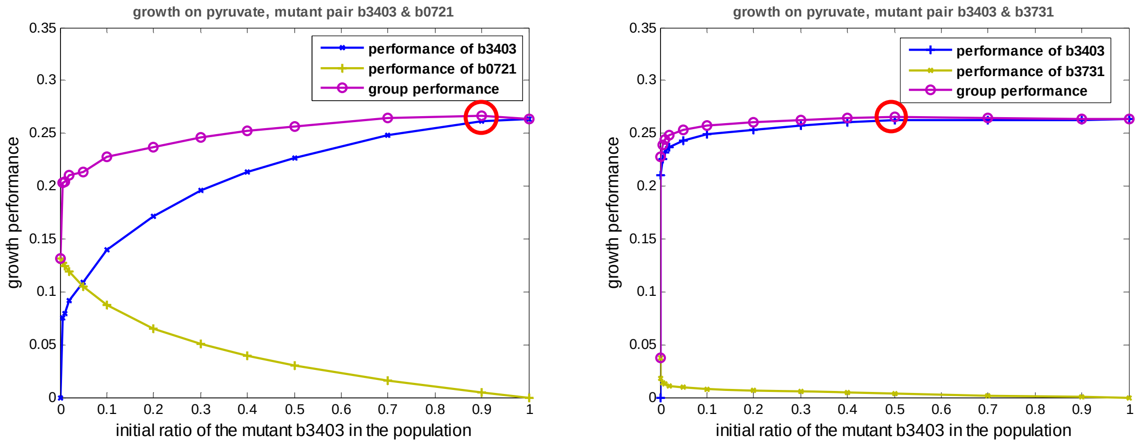

Figure 2.4 (Left) The growth performance of the mutants of b3403 and b0721 as they co-grow on limited *pyruvate* for various initial population ratios. The two mutants, b3403 and b0721 in equal initial frequency exhibit no relative benefit, since the growth performance of the group is below the performance of the mutant of b3403. However, as the population of the mutant of b3403 increases a benefit is observed (indicated with a red circle). (Right) The growth performance of the mutants of b3403 and b3731 as they co-grow on limited *pyruvate* for various initial population ratios. This mutant pair is beneficial when the mutants are initially in equal frequencies.

Multi-competitor growth

The growth of all the strain communities of each possible size has been simulated. Beneficial communities are observed. The percentages of the communities exhibiting either absolute or relative benefits among all the communities that are examined are shown separately for each community size in **Fig. 2.5**. Analysis of these strain compositions based on the flux and growth rate time profiles reveals that in all cases metabolic interactions are involved. The involved metabolic interactions of the most efficient strain triplet consisting of the mutants b2903, b0721 and b0721 (1.4% absolute benefit) and the most efficient strain pair mentioned previously, which consists of the mutants b3731 and b3403 (0.8 % absolute benefit) are shown in **Figure 2.6**. The exchange flux rate time profiles of *glycine* and *glycolate* involved in the efficient strain triplet and their effect on the maximum growth rates of the mutants b3403 and b0721, are shown in **Figure 2.7**.

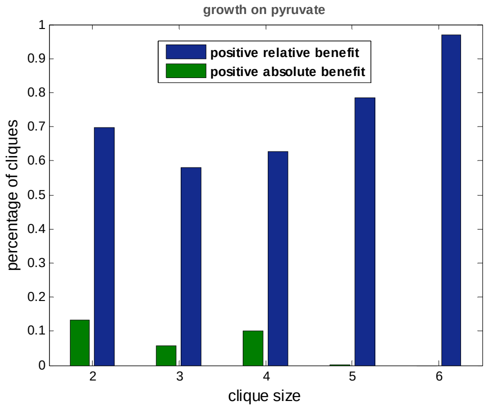

Figure 2.5 The percentage of strain communities exhibiting either relative or absolute benefit is shown. Absolute benefit implies relative benefit. Therefore, the communities with absolute benefit are part of the communities with relative benefit.

A subset of the highly central mutants and specifically those that corresponds to the knockout of the gene b0721, b4015, b2276 and b3731, is observed to play a beneficial role (relative benefit >0) in most of the pair interactions in which each of them participates (**Fig. 2.2**). Actually these mutants produce metabolites such as *acetate* and *formic acid*, which however are incapable of consuming (exception is the mutant of b2276, which is observed to consume the *formic acid* it produces). In their metabolic interactions, these mutants play the role of a provider of the specific nutrients, which are proved essential for the growth of the other members of the community. As expected when these mutants coexist with each other and since none of them is capable of consuming the available nutrients no benefit is observed. The unexplored mutant pairs are incapable to develop cross-feeding interactions but have been introduced in the diversity graph because in larger communities are observed to play an important and novel role since they allow another strain from the community to (beneficially) exploit the available products. One such example is the triplet b0721, b4015 and b0728 exhibiting relative benefit equal to 12.3%. Interestingly, all the involved pair-wise coexistences exhibit negative relative benefit. The cross-feeding interactions that occur are shown in Figure 2.8.

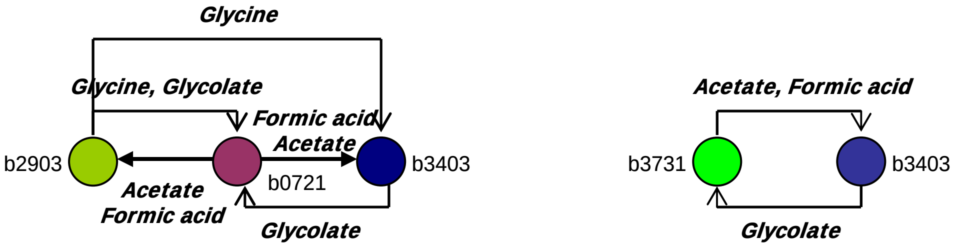

Figure 2.6 (Left) The strain triplet of b2903, b3403 and b0721 exhibits the maximum absolute benefit (1.4%) over all the simulated communities growing on *pyruvate*. The arrows show the metabolites that are exchanged during growth. (Right) The strain pair b3731 and b3403 exhibits absolute and relative benefit equal to 0.8% when co-grows on limited *pyruvate*. The metabolites that are exchanged between them are shown.

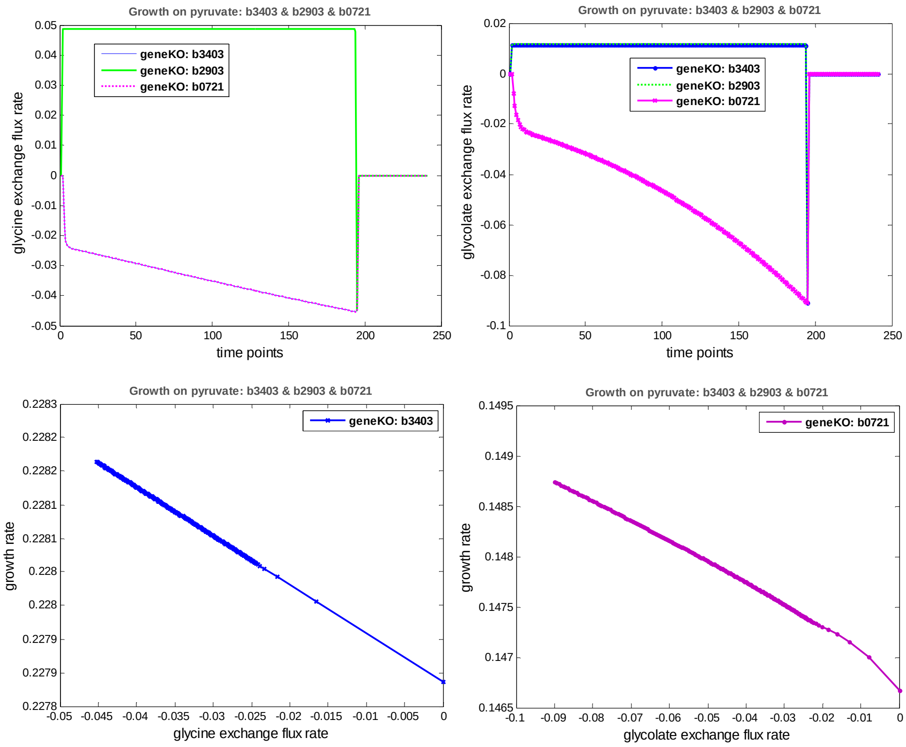

Figure 2.7 (Top) The exchange flux rate time profiles of *glycine* and *glycolate* for the three mutants that are derived from the single gene knockouts of b3403, b2903 and b0721 when co-grow on limited

pyruvate. (Bottom) The dependence of the growth rate during the uptake of a certain metabolite with respect to the flux rate of glycine for the mutant of b3403 (left) and the flux rate of glycolate for the mutant of b0721 (right).

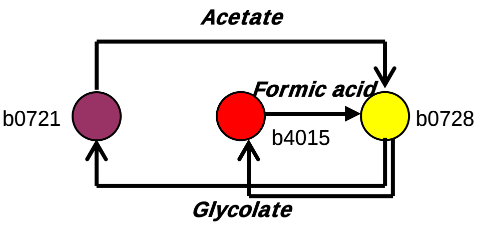

Figure 2.8 The cross-feeding interactions observed in the triplet consisting of the mutants b0721, b4015 and b0728 when grows on *pyruvate*. The pair b0721 and b4015 does not interact and exhibits rel. benefit equal to -7.5%. The pair b0721 and b0728 exhibits rel. benefit equal to -1.5% and the pair b4015 and b0728 exhibits rel. benefit equal to -4.7%. However, the triplet exhibits relative benefit equal to 12.3%.

Each community is comprised of smaller sub-communities, whereas the smallest size communities correspond to pairs. In **Figure 2.9** (Left), it is shown how the unexplored mutant pairs introduce non-linearities in the growth performance predictions of the larger communities when prediction is based on knowledge of the performance of the constituent pairs. Nevertheless, when the constituent triplets are considered (introduction of effective weights) for the prediction of larger communities instead of the pair-wise interactions, the correlation coefficient is significantly (**Table 2.1**) high (p-value = 0). All p-values in **Table 2.1** equal to 0 unless it is differently indicated. **Figure 2.9** (right) shows an example of the relation between the simulated performance and its prediction, which is based on pairs (depicted with a blue color) and on triplets (depicted with a cyan color).

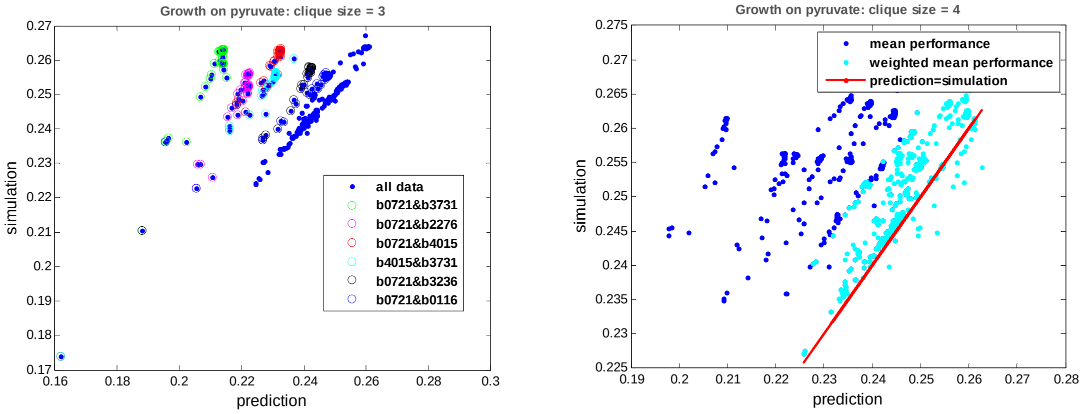

Figure 2.9 (Left) The simulated growth performance of all the triplets that are identified in the diversity graph of *pyruvate* with respect to the mean performance of the pair-wise interactions (prediction) is shown. A subset of the edges between the highly central mutants as participate in the triplets is also shown. (Right) The simulated growth performance of all the cliques of size 4 with respect to the predicted performance, which is estimated by the mean performance of the performances of the pair-wise interactions that each cliques consists of (blue color) or by the weighted mean performance where the effective weights are introduced (cyan color) for certain interactions.

Table 2.1: Growth on *pyruvate* – Correlation coefficient between predicted and simulated group performance

**Clique size:**	3	4	5	6
**R (based on pairs):**	-0.0328 (p-value=0.066)	-0.2404	-0.3585	-0.6206
**R (based on triplets):**	0.8325	0.8278	0.8320	0.9529
**Number of cliques:**	3130	3487	1822	364

3. Growth on *Glucose*

Multi-competitor growth

The growth of all the different mutant compositions has been evaluated. Absolute benefit is not observed (**Fig. 3.1**) in any clique of any size, which means that no bacterial community consisting of metabolically different, single-gene knockout mutants has been found to exploit better the given environment than the monoclonal populations. This observation implies that within the pool of single-gene knockouts, certain mutants such as the mutant cells of the deleted gene b0114 or the wild-type cells are observed to be capable of best exploiting the given environment when growing as monocultures. The mutant of the deleted gene b0114 exhibits end-point biomass, which is 0.2% higher than the performance of the wild-type in the same initial growth conditions. This mutant is observed to produce relatively less *acetate*, while it is not capable of by-producing *glycolate*. On the other hand, several mutant compositions are observed to exhibit positive relative benefit. The environmental-invariant mutant of the deleted gene b0721 produces *acetate*, *glycolate* and *formic acid* when grows on limited *glucose,* metabolites, which apart from *glycolate* it is not capable of consuming. In communities, this peculiar mutant thus plays the role of a provider of essential metabolites for growth having a purely altruistic behavior. The maximum relative benefit among the mutant pairs corresponds to the pair b0721 and b2779 and equals to 20% (**Fig.3.2**). The gene b2779 encodes the *enolase* and participates in the *Glycolysis Gluconeogenesis* pathway. *Acetate* and *formic acid* are also by-produced by the mutant of the deleted gene b2779, but no *glycolate*. The mutant of b2779 exploits all the available nutrients and it is observed that during the consumption of *glycolate* its growth rate increases as the uptake flux rate increases (**Fig.3.3**).

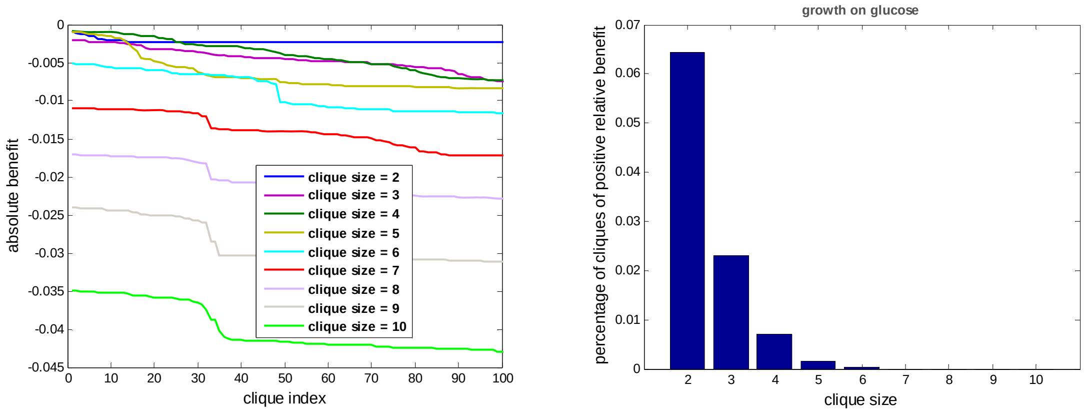

Figure 3.1 (Left) Absolute benefit is not observed (negative value) in any clique of any size. (Right) The percentage of strain communities exhibiting relative benefit is shown.

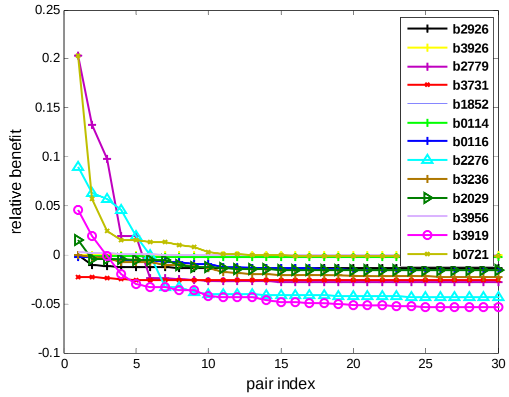

Figure 3.2 The relative benefit of the mutant pairs for growth on *glucose*. Each mutant pair corresponds to the potential interaction between a highly central mutant and another mutant in the diversity graph. The most beneficial mutant pair corresponds to b0721 and b2779 (20%). Both mutants are highly central.

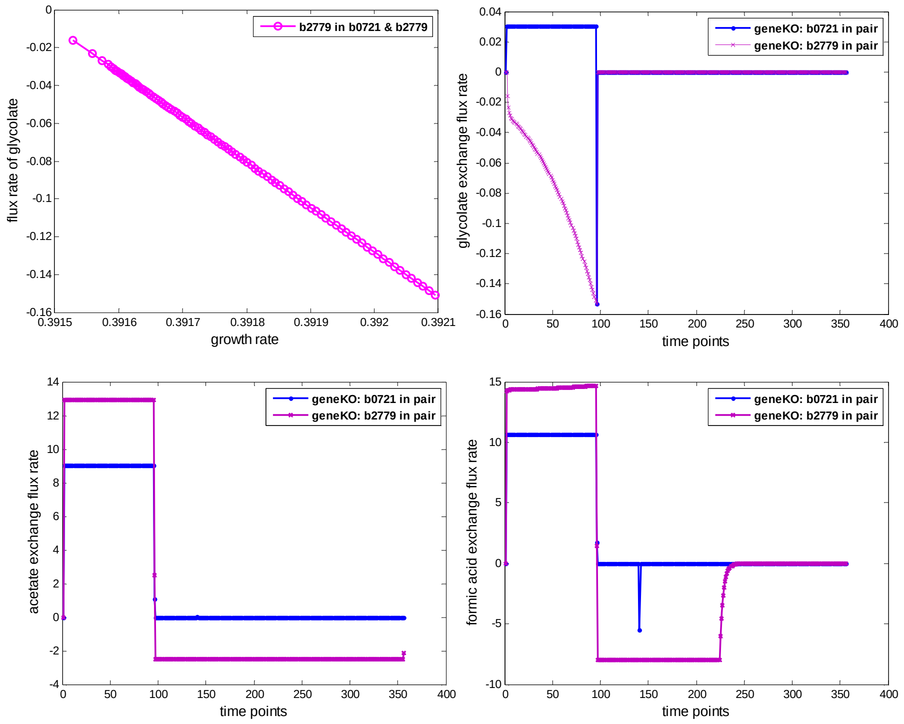

Figure 3.3 The growth properties of the mutant pair b0721 and b2779 when grows on glucose. The mutant b0721 is a pure altruist providing *glycolate*, *acetate* and *formic acid* to the mutant b2779, while being incapable of consuming them.

It is observed that knowledge of the growth performance of the mutant pairs is sufficient to accurately predict the group performance of larger communities (**Table 3.1**). Significant (p-value = 0) high correlation coefficient is observed between the predicted and the simulated growth performances. Nevertheless, knowledge of the mutant triplets improves the predictability when the communities are of size large enough (**Table 3.1**) as the significant (p-value = 0) high correlation coefficient show. All p-values in **Table 3.1** equal to 0. In **Figure 3.4** it is shown that the maximum divergence from the mean performance corresponds to cliques where unconstrained edges (see definition in **Methods**) are present.

Table 3.1: Growth on *glucose* – Correlation coefficient between predicted and simulated group performance

**Clique size:**	3	4	5	6	7	8	9	10
**R (based on pairs):**	0.8486	0.8208	0.8165	0.8062	0.8016	0.8061	0.8259	0.7540
**R (based on triplets):**	0.8799	0.9126	0.9283	0.9357	0.9323	0.9234	0.9145	0.9027
**Number of data (cliques):**	2087	6321	12229	15589	13077	6962	2136	288

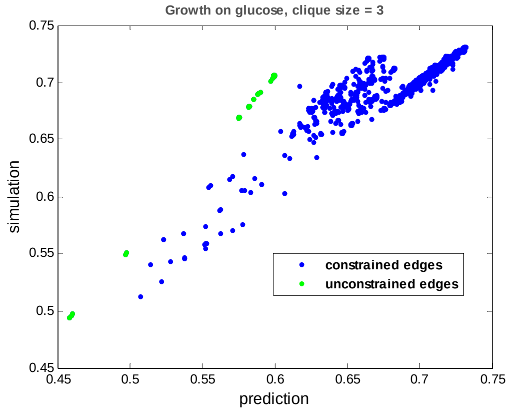

Figure 3.4 The simulated growth performance of all the triplets that are identified in the diversity graph of *glucose* with respect to the mean performance of the pair-wise interactions (prediction). The mutant triplets consisting of constrained edges are indicates with blue color. The complementary triplets that include unconstrained edges are shown with green color.

4. Growth on *Melibiose*

Single-growth characteristics

During growth on limited *melibiose* a number of secondary metabolites are observed to be by-produced by the mutants such as *acetate*, *dihydroxyacetone*, *ethanol*, *formic acid*, *fumarate*, *glycerol*, *glycolate* and *succinate*. Among all single-gene knockout mutants a subset was found to perform better than the wild-type cell under the same initial growth conditions. Maximum single growth performance exhibits the mutant of the deleted gene b1602. This gene of ERI value equal to 0.44 encodes the *NAD transhydrogenase* and the *NAD P transhydrogenase* participating in the *Oxidative Phosphorylation* pathway. Its high growth efficiency lies in its ability to efficiently catabolize the *melibiose*, the main carbon source and its combined high efficiency to metabolize *ethanol* among other metabolites such as *acetate* and *formic acid*. This mutant is also a highly central mutant in the diversity graph of *melibiose*.

Multi-competitor growth

Absolute benefit is not observed in any clique of any size indicating that there isn't strain community capable of exploiting better the given environment than the efficient mutant b1602. Nevertheless, relative benefit is observed (**Fig. 4.1**). The maximum relative benefit (~7%) is observed by the mutant pair 'b3731' and 'b2779'. The involved metabolic interactions of this beneficial pair are shown in **Figure 4.2**. The mutant b3731 produces *acetate*, *formic acid* and *ethanol.* It is observed to produce the maximum concentration of *acetate* among all mutants. However, it is incapable of consuming the produced *acetate* and *formic acid*. The mutant b2779 is capable of exploiting the available *formic acid* and partly the *acetate*. Thus, when the two strains coexist the mutant b3731 is mainly a provider of essential products to the mutant b2779. The triplet consisting of the mutants b2926, b2276 and b2779 exhibits the same relative benefit (~7%).

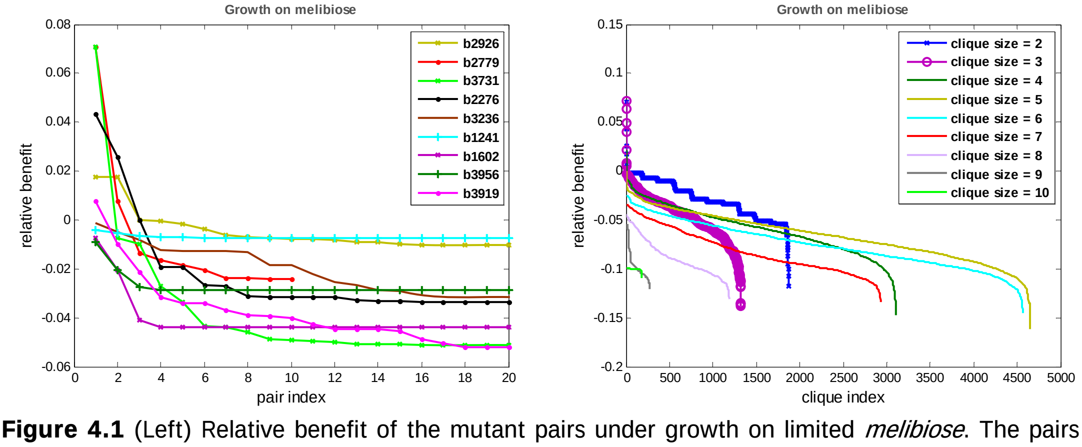

Figure 4.1 (Left) Relative benefit of the mutant pairs under growth on limited *melibiose*. The pairs of each highly central mutant are shown. (Left) Relative benefit of each clique. Relative benefit is not observed in any clique of size greater than five.

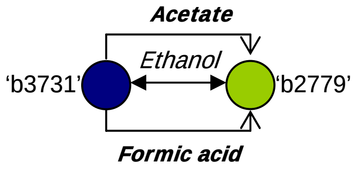

Figure 4.2 The arrows show the metabolites that are exchanged when the strain pair 'b3731' and 'b2779' is growing on limited *melibiose*. The mutant 'b3731' provides acetate and formic acid to the mutant 'b2779'. The red arrows correspond to *ethanol,* a metabolite which both mutants are capable of metabolizing.

It is observed that knowledge of the growth performance of the mutant pairs is sufficient to accurately predict the group performance of larger communities. Significant (p-value = 0) high correlation coefficient is observed between the predicted and the simulated growth performances (**Table 4.1**). However, unexploited pairs such as the mutants of b3731 and b2926 and the mutants of b3919 and b2926, which are incapable of fully exploiting the given environment and specifically of consuming *acetate* and *formic acid*, essential products for growth, exist causing divergent of the prediction from the simulated performance. Thus, an improvement in the correlation coefficient can be observed when prediction is evaluated based on simulated triplets. All p-values in **Table 4.1** equal to 0.

Table 4.1: Growth on *melibiose*– Correlation coefficient between predicted and simulated group performance

**Clique size:**	3	4	5	6	7	8	9	10
**R (based on pairs):**	0.8878	0.8854	0.8573	0.8916	0.9092	0.9190	0.9350	0.9034
**R (based on triplets):**	0.8794	0.8901	0.8789	0.9166	0.9217	0.9290	0.9178	0.8387
**Number of data (cliques):**	1322	3108	4648	4564	2934	1187	273	181

5. Growth on *Acetate*

Growth on *acetate* produces a diversity graph of star topology with only one mutant to be different from the rest mutants of the graph including the wild type cell. This one, central mutant is the mutant b2978 and is observed to be capable of by-producing a few amount of *glycolate* contrary to the rest mutants. However, it is not capable of consuming the *glycolate* it produces. The gene b2978 encodes the enzyme *glycolate oxidase* and participates in the *alternate* carbon metabolism subsystem. The growth performance of the cells when this gene is deleted is not considerably altered with respect to the performance of the wild-type cells. When the mutant b2978 coexists with any other mutant, *glycolate* exchange is observed according the flux rate profiles. However, no effect on the growth performance of the population from this exchange is observed. The absolute benefit is consistently negative and the relative benefit is negative apart from few cases such as the mutant pair b2978 and b3945, which exhibits 0.1% benefit.

6. Growth on *Glycine*

Growth on *glycine* produces a simple diversity graph where two central mutants are identified. The fist is derived from the deletion of the metabolic gene b2276, which is involved in the *Oxidative Phosphorylation* pathway. This mutant has reduced growth performance compared to the growth performance of the wild-type cell. It is observed that while the other mutants as well as the wild-type cell produce *formic acid,* the specific mutant is incapable of. The second central mutant corresponds to the gene b2978. When this gene is deleted the cell produces *glycolate* contrary to the other mutants. When the mutant b2276 coexists with any other mutant relative benefit is observed (**Fig. 6.1**). Relative benefit is also observed when the two central mutants b2276 and b2978 co-grow. The mutant of the deleted gene b2276 exploits the by-product *formic acid* that is provided in the environment by the partner mutant (or the wild-type) and increases its growth rate (**Fig. 6.2**). The same benefit is also observed between b2276 and b2978. In other words, the mutant of the deleted gene b2276 is an exploiter, a strictly consumer of the secondary metabolite *formic acid* which another mutant by-produces and provides in the common medium under co-growth on limited *glycine*.

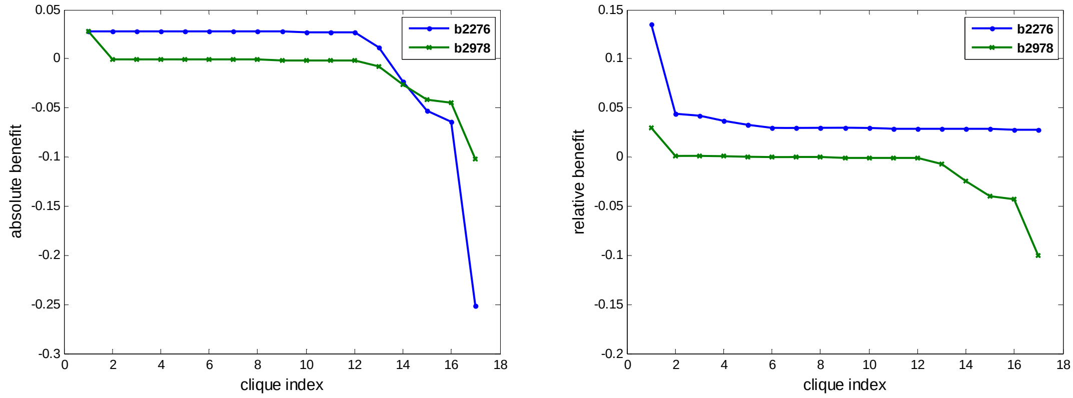

Figure 6.1 The absolute and relative benefit separately for the two central mutants that appear in the diversity graph of growth on limited *glycine*.

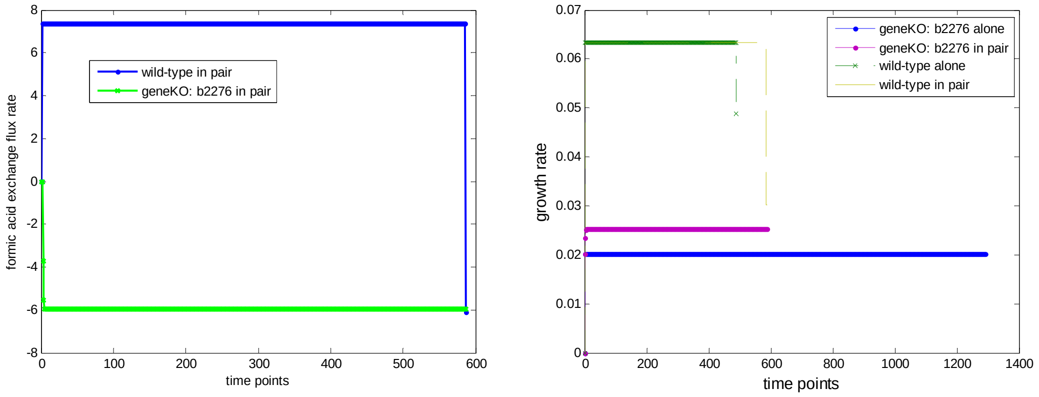

Figure 6.2 The co-growth of the wild-type cell with the mutant of the deleted gene b2276 in a common environment where *glycine* is provided as the main carbon source. The wild-type cell produces *formic acid* while only the mutant is capable of consuming it (Left). The exchange of the novel -for the mutant- metabolite *formic acid* increases its growth rate (Right).

Exhaustive Analysis of the WT-KO$^-$ pairs in different carbons

Table A (updated from [1]) summarizes the conditions in which the co-growth of a specific strain with the wild type cell shows superior group performance. The relative yield ($Yield_{rel}$) is calculated with respect to the performance of the homogeneous population of either the wild type or the participating mutant ($gene_{S\_KO}$) depending on which of the two participant's performance is maximum. The absolute yield ($Yield_{abs}$) is calculated with respect to the mutant ($gene_{\_KO}$) of the best homogeneous performance in the certain environment.

TABLE A

CONDITIONS OF SUPERIOR GROUP PERFORMANCE – GROWTH ON CARBON SOURCE

CARBON SOURCE	HOMOGENEOUS POPULATION							HETEROGENEOUS POPULATION			
	WT	Best KO$^-$			Best synergistic KO$^-$			Synergy			
	$BM_{\_WT}$	$gene_{\_KO}$	BM	$Yield_{WT1}$ (%)	$gene_{S\_KO}$	BM	$Yield_{WT2}$ (%)	BM_{S_WT}	BM_{S_KO}	$Yield_{rel}$ (%)	$Yield_{abs}$ (%)
glyclt	0.1025	-	-	-	'b2276'	0.0944	**-7.95**	0.085116	0.025721	**8.07**	**8.07**
gly	0.0660	-	-	-	'b2276'	0.0399	**-39.4**	0.06132	0.006615	**2.89**	**2.89**
cit	0.3585	'b0331'<br>'b0333'<br>'b0334'	0.3603	**0.49**	'b0728'	0.3331	**-7.08**	0.23057	0.1389	**3.05**	**2.54**
pyr	0.2365	'b3403'	0.2417	**2.19**	'b0721'	0.1313	**-44.8**	0.217158	0.028121	**3.71**	**1.48**
ser_D	0.2375	'b3403'	0.2433	**2.44**	'b0721'	0.1362	**-42.6**	0.218683	0.027194	**3.53**	**1.06**
arg_L	0.6223	'b1744'	0.6544	**5.16**	'b1744'	0.6544	**5.16**	0.36028	0.30053	**0.97**	**0.97**
4abut	0.5206	'b1849'	0.5211	**0.09**	'b0451'	0.3982	**-23.5**	0.39198	0.13054	**0.36**	**0.27**
melib	1.3837	'b1602'	1.4142	**2.20**	'b2276'	1.0878	**-21.4**	1.1073	0.28443	**0.58**	**-1.58**
tre	1.3883	'b1602'	1.4172	**2.08**	'b1241'	1.3788	**-0.68**	0.76453	0.62803	**0.31**	**-1.74**
sucr	1.3883	'b1602'	1.4172	**2.08**	'b1241'	1.3788	**-0.68**	0.76453	0.62803	**0.31**	**-1.74**
malt	1.3883	'b1602'	1.4172	**2.08**	'b1241'	1.3788	**-0.68**	0.76453	0.62803	**0.31**	**-1.74**
mnl	0.7782	'b1602'	0.7965	**2.34**	'b2276'	0.6283	**-19.2**	0.65929	0.12161	**0.34**	**-1.95**
akg	0.3933	'b4015'	0.4147	**5.44**	'b0721'	0.1343	**-65.8**	0.32267	0.078385	**1.97**	**-3.29**
glu_L	0.4764	'b4015'	0.5089	**6.83**	'b3236'	0.4774	**0.21**	0.26654	0.21778	**1.66**	**-4.83**

Edge definitions

Additional properties that may determine the edges of the diversity graphs in different ways concern the ability of the involved strains to consume the byproducts.

Constrained: The metabolic difference is taken only upon byproducts that at least one of the two potential participants is capable of consuming. Novel byproducts for one of the two potential participants are constraint-free. This condition on the edges of the graph implies that any two metabolically different strains also have the potential to exchange nutrients (metabolically interact).

Unconstrained: The 'consumability' constraint is relaxed. The relaxed condition implies that two strains are allowed to both provide a certain nutrient to the public pool without consuming it, where however, a third strain of different metabolic capabilities of both the two strains can exploit. Thus, the metabolic difference between two strains might be important in a divergent community, even if it does not lead to direct exchange of nutrients.

References

1. Tzamali E, Reczko M: **The benefit of cooperation: Identifying growth efficient interacting strains of Escherichia coli using metabolic flux balance models**. *8th IEEE International conference on bioinformatics and bioengineering, GREECE* 2008.
